# Development of canine parvovirus-2-based recombinant pseudoviruses expression system: a potential vaccine platform

**DOI:** 10.1186/s13567-026-01789-9

**Published:** 2026-06-18

**Authors:** Bichen Miao, Qian Du, Liu Yang, Jin Yan, Fukang Tu, Yiyuan Jiang, Ning Xu, Songbiao Chen, Yong Huang, Dewen Tong

**Affiliations:** 1 College of Veterinary Medicine, Northwest A&F University, Yangling, China; 2 Engineering Research Center of Efficient New Vaccines for Animals, Ministry of Education, Yangling, China; 3 Key Laboratory of Ruminant Disease Prevention and Control (West), Ministry of Agriculture and Rural Affairs, Yangling, China; 4 Engineering Research Center of Efficient New Vaccines for Animals, Universities of Shaanxi Province, Yangling, China

**Keywords:** CPV-2, CDV, pseudovirus, vaccine, immune response

## Abstract

Canine parvovirus-2 (CPV-2) and canine distemper virus (CDV) are two highly infectious and pathogenic pathogens that harm canids and various carnivores, and often cause co-infections in clinical settings. Although commercial attenuated live vaccines against CPV-2 and CDV have been widely used to prevent these viral infections, there are still issues of biosafety and insufficient protection against new variants, thus requiring novel vaccines. In this study, we developed a recombinant pseudovirus expression system based on the CPV-2 full-length infectious clone, and found that the recombinant CPV-2 pseudovirus expressing CDV H protein could efficiently protect dogs against both CPV-2 and CDV infections. We first designed a recombinant pseudovirus vector based on the CPV-2 backbone and established a stable cell system for the production of CPV-2 recombinant pseudoviruses carrying foreign genes. These pseudoviruses preserved the morphology and particle size of native CPV-2, while maintaining hemagglutination activity against porcine erythrocytes, and demonstrated the ability to effectively infect permissive cells to express the harbored foreign gene. Next, we produced a recombinant CPV-2 pseudovirus expressing CDV H protein, and the CPV–CDV recombinant pseudovirus induced effective cellular and humoral immune responses in dogs. The serum from CPV–CDV immunized dogs could effectively neutralize CPV-2 and CDV infections in susceptible cells. Importantly, CPV–CDV completely protected dogs against challenge with CPV-2 and CDV. In summary, we have successfully developed a stable production system of recombinant CPV-2 pseudoviruses, which has great potential for development as a vaccine platform.

**Supplementary Information:**

The online version contains supplementary material available at 10.1186/s13567-026-01789-9.

## Introduction

Canine parvovirus-2 (CPV-2) and canine distemper virus (CDV) are highly infectious and pathogenic to canids and various carnivores, and these two viruses usually co-infect in clinic samples, posing significant threats to the health of susceptible animals [1, 2]. Particularly in wildlife populations, a single outbreak of epidemics may cause population collapse or even local extinction [3, 4]. Attenuated live vaccines and inactivated vaccines have been widely used to prevent and control CPV-2 and CDV infection-induced diseases. Inactivated vaccines offer a high safety profile, but low immunogenicity, potentially resulting in insufficient protective efficacy [5]. For attenuated live vaccines, there have been reports that attenuated vaccine strains of both CPV-2 and CDV have residual virulence, and thus pose health threats to vaccinated wildlife [6, 7]. This is all the more remarkable that the administration of attenuated live vaccines can lead to the transmission and spread of viruses in the natural environment, and there is the risk of vaccine strains undergoing virulence reversion [8, 9]. Furthermore, in recent years, new variants of CPV-2 and CDV have emerged continuously, exhibiting changes in antigenicity, immunogenicity, and virulence [10–12]. The antigenic differences between vaccine strains and antigenic variants may reduce the efficacy of commercially available vaccines on the basis of the original CPV-2 and CDV strains, and the vaccination failure is largely related to inadequate protection against new antigen variants [13, 14]. Therefore, it is crucial to use the prevalent virus strains to prepare genetic engineering vaccines. It is still necessary to develop a safer and more effective novel vaccine to prevent both CPV-2 and CDV [5, 15, 16].

The family *Parvoviridae* have emerged as valuable gene delivery platforms in the fields of gene therapy and recombinant protein production. These could be categorized into two groups: adeno-associated virus (AAV) and autonomous parvoviruses (APVs) [17]. At present, AAV has been developed into safe and effective vehicles for the long-term stable expression of therapeutic genes, and multiple gene therapy drugs based on recombinant AAV (rAAV) have been approved for the treatment of rare diseases [18]. On the other hand, the typical representatives of APVs vectors include the minute virus of mice (MVM), H-1 parvovirus (H-1PV), and LuIII parvovirus. These viruses are capable of effectively infecting a variety of human cell lines and mediate the transient and efficient expression of exogenous genes, as such demonstrating great potential in therapeutic strategies that require transient gene expression [19–22].

The vectors of the family *Parvoviridae* have attracted widespread attention in gene delivery systems owing to their unique biological properties. Their viral particles are small in size (with a diameter of approximately 20–26 nm), and the genetic architecture is simple (with a single-stranded DNA genome length of about 4–6 kb) [23]. Both the genome and capsid proteins exhibit high plasticity, through genetic engineering strategies: the reverse terminal repeats (ITRs) necessary for viral packaging was retained, and part or all of the viral coding sequence was replaced with the gene of interest, thereby achieving precise insertion of the target heterologous gene sequence [18, 24, 25]. CPV-2 is a member of the APVs with a single-stranded, negative-sense DNA genome of approximately 5000 nt encapsulated in the icosahedral particle of 60 subunits of structural proteins [26]. The CPV-2 genome contains two open reading frames (ORFs; ORF1 encoding nonstructural proteins NS1 and NS at the 5′end, and ORF2 encoding structural proteins VP1 and VP2 at the 3′ end), as well as noncoding regions formed by inverted terminal repeats (ITR) regions at both ends [26]. The VP2 coding sequence is completely contained in VP1 sequence and shares the same 3′ end with VP1, while VP1 has a unique sequence encoding 227 residues at the N-terminal, known as the VP1 unique region (VP1u), which has been reported to play an important role during viral cell entry and endosomal trafficking [26, 27].

Our previous study successfully constructed the reverse genetic system of CPV-2 to enable manipulation of the virus at the genetic level [28]. In this study, we first constructed a recombinant CPV-2 pseudovirus plasmid containing EGFP sequences at VP2 encoding region, as well as the packaging plasmids expressing CPV-2 VP1 and VP2. These plasmids were used to obtain the recombinant CPV-2 pseudovirus expressing EGFP. Further, we generated a HEK293 cell line to stably express CPV-2 VP1 and VP2, and this cell line could be used to produce recombinant CPV-2 pseudoviruses. Subsequently, we produced a recombinant CPV-2 pseudovirus expressing CDV H protein, which could effectively induce host immune responses against both CPV-2 and CDV in dogs and protect them from the infectious challenges. These results indicate that the recombinant CPV-2 pseudovirus expression system we generated in this study has the potential to be a powerful tool for the development of novel vaccines.

## Materials and methods

### Plasmid construction

The full-length infectious clone plasmid pX-CPV-2c has been constructed in our laboratory as previously reported. The coding sequences of VP1 and VP2 were amplified with primers P1/P2 (P1: 5′-GAGCTAGCGGGGGGGTTGGTGTGT-3′ and P2: 5′-CGGCTGCAGCTAGTATAATTTTCTTGG-3′) and P3/P2 (P3: 5′-TTCGAGACGACTTGGATTAAGACTTGTGCCTCCAGGTTATAAAT-3′) designed according to the CPV-2 sequence (MT892649), and the 5′UTR sequence of CPV-2 VPs was also amplified by polymerase chain reaction (PCR) (F1: 5′-CCGCTCGAGGCAAAGACTTAGAGACACAAGCGG-3′, R1: 5′-CTCTCCTGGCTCTCTTTGCC-3′, and F2: 5′-CCGCTCGAGGCAAAGACTTAGAGACACAAGCGG-3′; R2: 5′-AATCCAAGTCGTCTCGAAAATCT-3′). Then the 5′UTR sequence fusion with VP1 or VP2 sequences were generated by overlap PCR, which were named 5′UTR-VP1 or 5′UTR-VP2; then these sequences were cloned into pCI-neo plasmids to generate pCI-VP1 and pCI-VP2. Afterward, the sequences of 5′UTR-VP1 and 5′UTR-VP2 were subcloned into pCDH-puro and pLVX-neo plasmids to produce recombinant pCDH-VP1-Puro and pLVX-VP2-Neo. The pCPV-EGFP plasmid was generated by inserting the EGFP coding sequence downstream of the start codon (ATG) in the VP2 coding region (ORF2) of pX-CPV-2c. All plasmids were constructed using standard methods and verified by sequencing.

### Generation of stable cell line expressing VP1 and VP2

pCDH-VP1-Puro and pLVX-VP2-Neo were transfected in HEK293 cells along with helper plasmids psPAX2 and pMD2.G, respectively, for the package of two types of lentiviral particles. The recombinant lentiviruses pCDH-VP1 and pLVX-VP2 were collected at 48 h post-transfection. Next, the recombinant lentivirus pCDH-VP1 was used to generate a HEK293 cell line (HEK293^*VP1*^) expressing CPV-2 VP1 that was consecutively screened using 3 μg/mL of puromycin for 10 generations. Subsequently, the recombinant lentivirus pLVX-VP2 was infected to the HEK293^*VP1*^ cells to generate a cell line (HEK293^*capsid*^) expressing both CPV-2 VP1 and VP2 using 400 μg/mL G418 for 10 generations.

### Indirect immunofluorescence assay

The experiment was performed using the F81 cell line (Cat. no.: 305015; Cytion), an epithelial-like cell line derived from feline kidney tissue. The cells were cultured in Dulbecco’s Modified Eagle’s Medium (DMEM) supplemented with 10% fetal bovine serum (FBS) at 37 °C in 5% CO_2_.

F81 cells were seeded on cell slides in 24-well plates, and infected with CPV-2 recombinant pseudovirus while growth to approximately 80% cell confluence. The infected cells were fixed by 4% paraformaldehyde, followed by permeabilization with 0.5% Triton X-100. The cells were blocked with phosphate-buffered saline (PBS) containing 2% nonfat milk for 2 h at room temperature, and then incubated with mouse anti-CPV-NS1 polyclonal antibody (prepared in our laboratory) at a dilution of 1:1000 for 8 h at 4 ℃. After washing three times with PBS, the cells were incubated with Alexa Fluor^®^ 594-conjugated goat anti-mouse antibody (Abcam, Cambridge, UK) at a dilution of 1:200 for 8 h at 4 ℃, followed by incubation with DAPI (4',6-diamidino-2-phenylindole) for 15 min at 37 ℃. Finally, the slides were observed by fluorescence microscopy.

The anti-CPV-NS1 polyclonal antibody utilized in this assay was prepared by immunizing female BALB/c mice (6–8 weeks old) with purified truncated CPV-NS1 fusion protein. Briefly, the fusion protein was expressed in *Escherichia coli* following the construction of the prokaryotic expression vector, and purified using His-tag Purification Resin. Mice received one primary immunization and two booster immunizations with the purified protein, and polyclonal antiserum was collected from the immunized animals. The antigenicity and specificity of this polyclonal antiserum have been confirmed in preliminary experiments.

### Western blot

The cells were harvested and lysed, and then centrifuged to gain the supernatant. The cell supernatants were boiled with 5 × sodium dodecyl sulfate (SDS) loading buffer, and separated in 12% SDS–polyacrylamide gels. The separated proteins were transferred to PVDF membranes. After blocking with 5% nonfat milk for 2 h at room temperature, the membranes were incubated with corresponding primary antibodies and HRP-conjugated secondary antibodies at appropriate dilution for 8 h at 4 ℃, and then detected using enhanced chemiluminescence (ECL).

### Enrichment of pseudoviral particles

The pseudovirus plasmid transfected cell lysis supernatants were pre-enriched using 20% sucrose solution with 10 000 *g* centrifugation at 4 ℃ for 5 min. Then the precipitates were resuspended in PBS to be further enriched using centrifugation of 50%, 35%, and 20% sucrose gradient at 4 ℃ 200 000 *g* for 4 h. The portion containing the pseudoviruses in the centrifuge tubes were collected and purified through gradient dialysis.

### Transmission electron microscopy (TEM) analysis

The suspended droplets of virus particles were absorbed on carbon-coated copper net, then negatively stained with phosphotungstic acid, and rinsed to remove excess impurities. The samples were air-dried and stored at room temperature. The morphologies of the virus particles were observed by TEM at an acceleration voltage of 100 kV, with a magnification of 80 000× .

### Enzyme-linked immunosorbent assay (ELISA)

The CPV-2 VP2 gene was cloned into pET-32a plasmid, and then the recombinant plasmid was transfected into *E. coli* (BL21). The expression of recombinant CPV-2 VP2 protein was induced with IPTG, and the expressed recombinant CPV-2 VP2 protein was diluted with 0.1 M bicarbonate buffer in pH 9.6 to coat 96-well ELISA plates. After being blocked with 1% bovine serum albumin (BSA) at 37 °C for 2 h, the coated 96-well plates were incubated with the dog serum samples diluted twofold serially with PBS in the range from 1:200 to 1:25 600 at 37 °C for 1.5 h. Then the plates were washed three times, and the bound antibodies were detected using HRP-labelled goat anti-dog immunoglobulin G (IgG) at 37 °C for 1.5 h.

### Serum neutralization assay

The serum samples were inactivated at 56 °C for 30 min and then serially twofold diluted with serum-free DMEM in the dilution range of 1:8 to 1:1024. The diluted serum samples were divided into two groups for neutralization assay against CPV-2 and CDV, respectively. For each assay, 50 μL of diluted serum samples was mixed with an equal volume of CPV-2 (100 TCID_50_/50 μL) or CDV (100 TCID_50_/50 μL), respectively, and incubated at 37 °C for 1 h. Subsequently, the serum–virus mixtures were inoculated into 96-well plates preseeded with F81 cells or Vero cells at a volume of 100 μL per well, and the plates were incubated under 5% CO_2_ at 37 °C for 96 h. The cytopathic effects (CPE) of the cells were observed. The highest serum dilution that could neutralize CPV-2 or CDV infections were considered as the neutralization endpoint, and the neutralizing antibody titers of the serum samples were calculated using the Reed and Muench method.

### Animal experiments

Twenty-five 4-week-old beagles were randomly divided into five groups. Three of these groups received intramuscular injection of CPV–CDV pseudovirus at doses of 1 × 10^6^ TCID_50_, 4 × 10^6^ TCID_50_, and 1.6 × 10^7^ TCID_50_ respectively, with the injection volume of 1 mL. One group was immunized with the 1 mL of commercial vaccine (Nobivac Puppy DP) manufactured by MSD Animal Health, which contains attenuated live CPV-2 (1 × 10^5^ TCID_50_) and CDV (1 × 10^7^ TCID_50_). The last group was injected with the same volume of PBS. All groups of dogs were immunized three times at 0, 3, and 6 weeks post-immunization (wpi), and then challenged with 1 × 10^6^ TCID_50_ CPV-2 and 1 × 10^7^ TCID_50_ CDV at 8 wpi. All the dogs were monitored daily in health status, and we take measures to ensure animal welfare.

### Ethics approval and consent to participate

All animal experimental procedures have been reviewed and officially approved by the Animal Ethics Committee of Northwest A&F University, China (permit 20231106). The experimental design and result reporting of this study strictly followed the ARRIVE 2.0 guidelines as described in Additional file 1. We clearly defined humane endpoints and the euthanasia protocol was formulated and strictly implemented. Throughout the post-challenge period, we monitored the health status of the experimental animals and provided supportive care measures. All efforts were made to minimize animal suffering.

### Statistical analysis

All the results were representative of three independent experiments. Data were presented as mean ± SEM (SD). The independent sample *t*-test was used for comparisons between two groups, and comparisons among groups were analyzed by ANOVA followed by the Bonferroni post hoc test or unpaired *t* tests. *P* values of < 0.05 or < 0.01 were considered as statistically significant.

## Results

### Construction of trafficking and packaging plasmids for recombinant pseudovirus based on CPV-2

**Figure 1:**
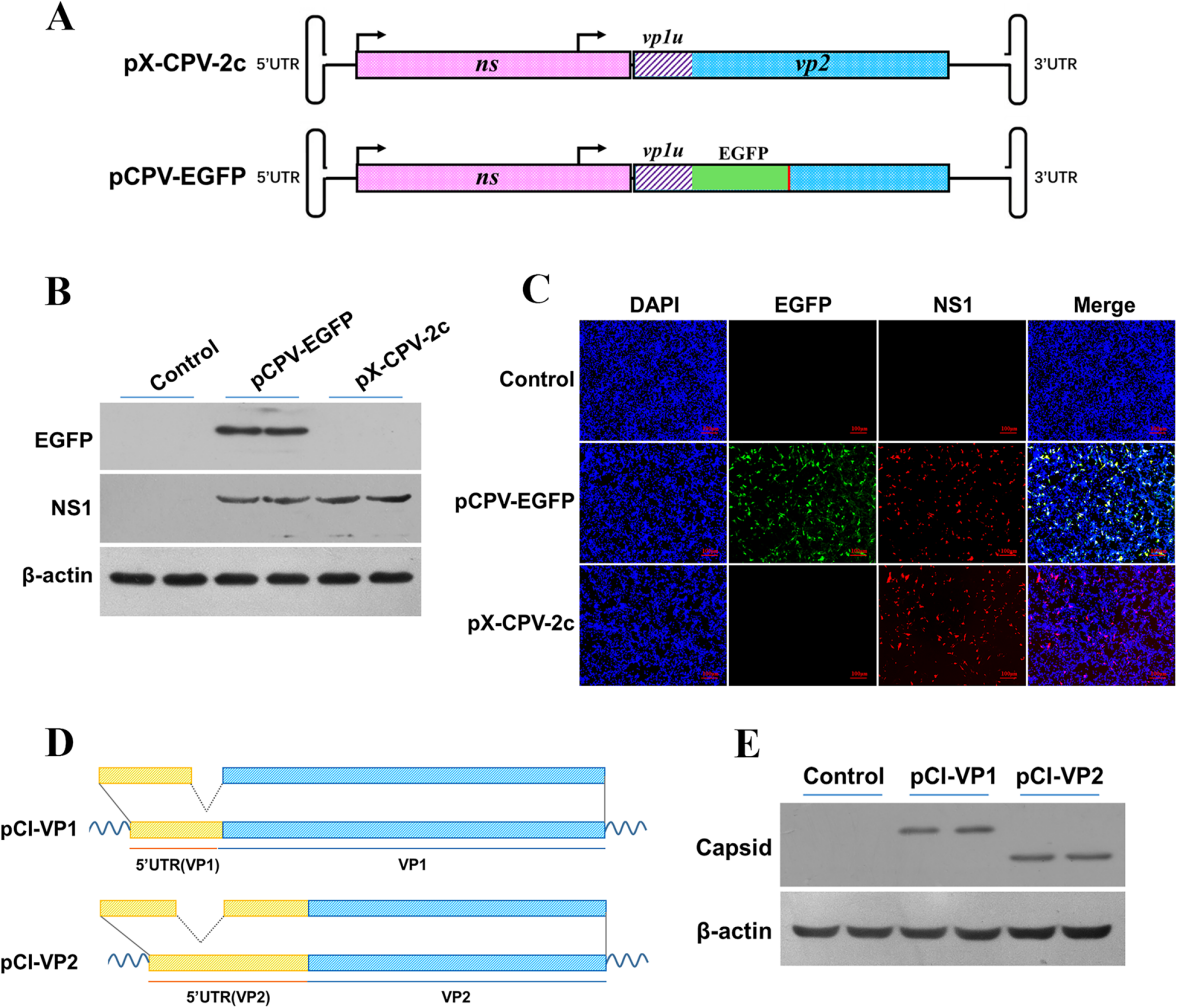
**Construction and expression of recombinant trafficking and packaging plasmids of CPV-2. A** Schematic diagram of CPV-2 recombinant plasmids. The VP2 sequence was replaced from the N-terminus with the EGFP gene sequence. **B, C** Western blot analysis **(B)** and Immunofluorescence assay **(C)** of the expression of EGFP and NS1 in CPV-EGFP transfected cells. **D** Schematic diagram of the expression vectors of VP1 and VP2 fused with 5′UTR sequences of CPV-2 capsids to their upstream, respectively. **E** Western blot analysis of VP1 and VP2 expression of pCI-VP1 and pCI-VP2 transfected cells.

We previously constructed a full-length infectious clone plasmid of CPV-2 (pX-CPV-2c) [28], to develop a trafficking system of exogenous gene, we replaced the VP2 coding region in the CPV-2 infectious cloning plasmid with EGFP gene (named as pCPV-EGFP, Figure 1A). To investigate if the pCPV-EGFP plasmid could successfully express the exogenous gene, we transfected pCPV-EGFP into HEK293 cells, and found that EGFP expression could be detected along with CPV-2 NS1 (Figure 1B, C). To achieve the self-assembly of the recombinant genome with wild-type capsid proteins to form progeny virus particles in vitro, we amplified the gene sequences of CPV-2 VP1 and VP2 that fused to the viral capsid 5′ untranslated region (5′UTR) sequence, which is essential for the effective expression of VP1 and VP2. The 5′UTR sequence was fused into the upstream of VP1 and VP2 gene using overlap extension PCR, respectively, and then the fused 5′UTR-VP1 and 5′UTR-VP2 were subcloned into eukaryotic expression plasmid pCI-neo to construct the recombinant plasmids pCI-VP1 and pCI-VP2 (Figure 1D). The control plasmid pCI-neo and the recombinant plasmids pCI-VP1 and pCI-VP2 were transfected into HEK293 cells respectively, and the effective expression of CPV-2 VP1 and VP2 were confirmed (Figure 1E). These results show that we had successfully constructed an exogenous EGFP gene transfer plasmid on the basis of the full-length infectious clone plasmid of CPV-2, as well as the packaging plasmids expressing CPV-2 VP1 and VP2.

### Production and identification of recombinant CPV-2 pseudovirus using the trafficking and packaging plasmids

**Figure 2:**
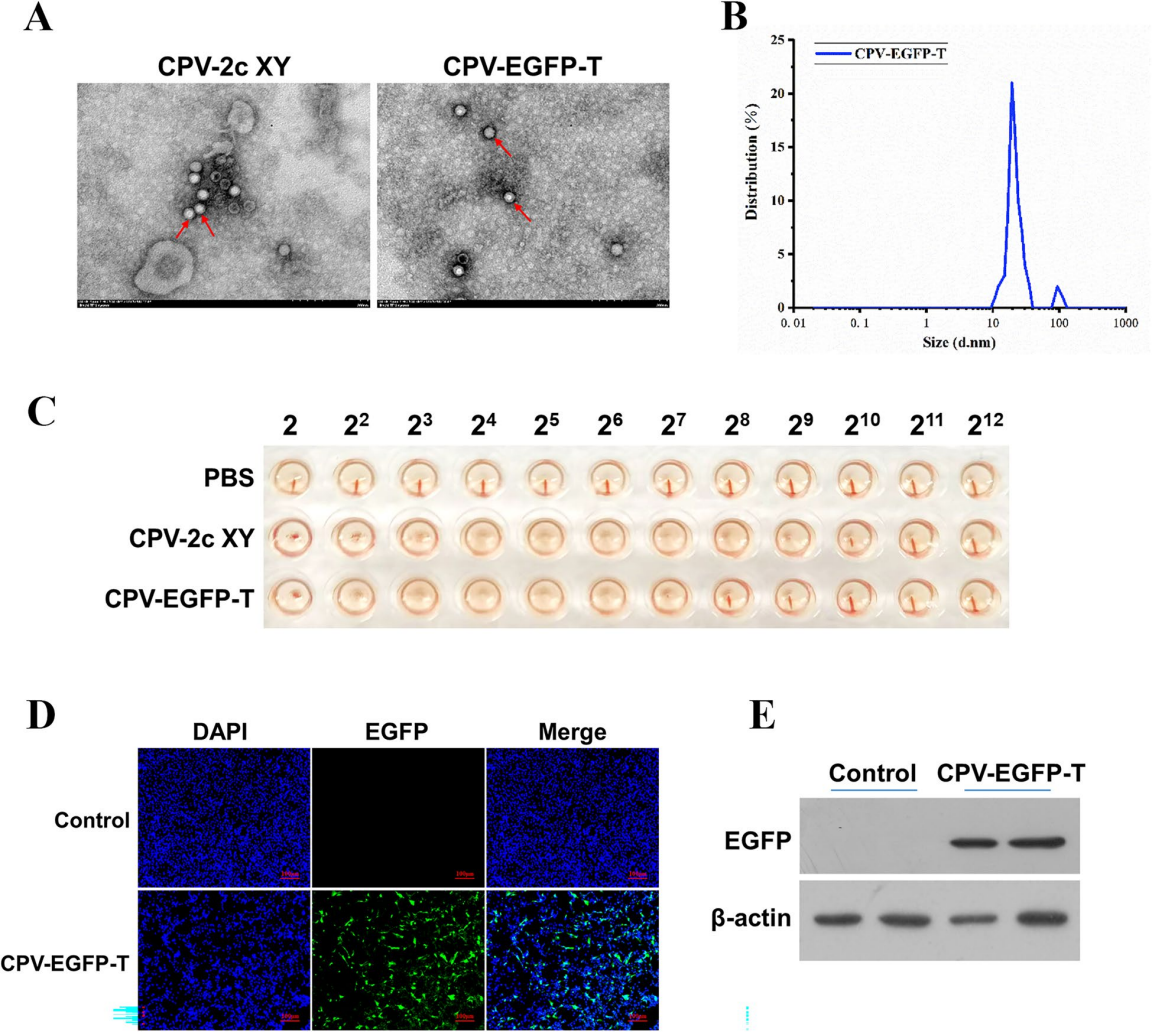
**Assembly analysis of CPV-2 recombinant pseudovirus generated by the trafficking and packaging plasmids. A** TEM analysis of CPV-EGFP-T assembly, scale bar = 200 nm. **B** The size distribution of CPV-EGFP-T was analyzed by DLS. **C** The characteristic of agglutinating pig erythrocytes of CPV-EGFP-T. **D, E** Immunofluorescence assay **(D)** and western blot analysis **(E)** of the expression of EGFP in CPV-EGFP-T infected F81 cells.

To determine if the exogenous EGFP gene transfer plasmid and the packaging plasmids expressing CPV-2 VP1 and VP2 could assemble CPV-2 recombinant pseudovirus in cells, we co-transfected these three plasmids (pCI-VP2, pCI-VP1, and pCPV-EGFP) into HEK293 cells at a mass ratio of 1:1:2. The supernatant of the co-transfected HEK293 cells was collected to detect the production of recombinant CPV-2 pseudovirus using transmission electron microscopy (TEM) and dynamic light scattering (DLS) analysis. The TEM results showed that the co-transfection of the three plasmids could effectively produce recombinant CPV-2 pseudoviruses (CPV-EGFP-T), which were a similar size to CPV-2 virions produced by the infectious clone plasmid pX-CPV-2c (Figure 2A). The DLS results showed that the diameter of the recombinant CPV-2 pseudoviruses was mainly in the range of 20–30 nm (Figure 2B). The CPV-EGFP-T was produced with a titer of 1 × 10^8.6^ TCID_50_/mL. Similar to the wild-type CPV-2, the CPV-EGFP-T maintained the ability to agglutinate porcine erythrocytes, and its agglutination titer was lower than that of wild-type CPV-2 (1 × 10^8^ TCID_50_/mL), as shown in Figure 2C. Further, we infected F81 cells with the recombinant pseudovirus CPV-EGFP-T, and the infected cells expressed high level of EGFP (Figure 2D, E). These results show that the co-transfection of trafficking and the packaging plasmids could effectively produce recombinant CPV-2 pseudovirus, which has the ability to infect the susceptible F81 cells and expressing exogenous EGFP gene.

### Construction of a stable cell line for the recombinant CPV-2 pseudovirus production

**Figure 3:**
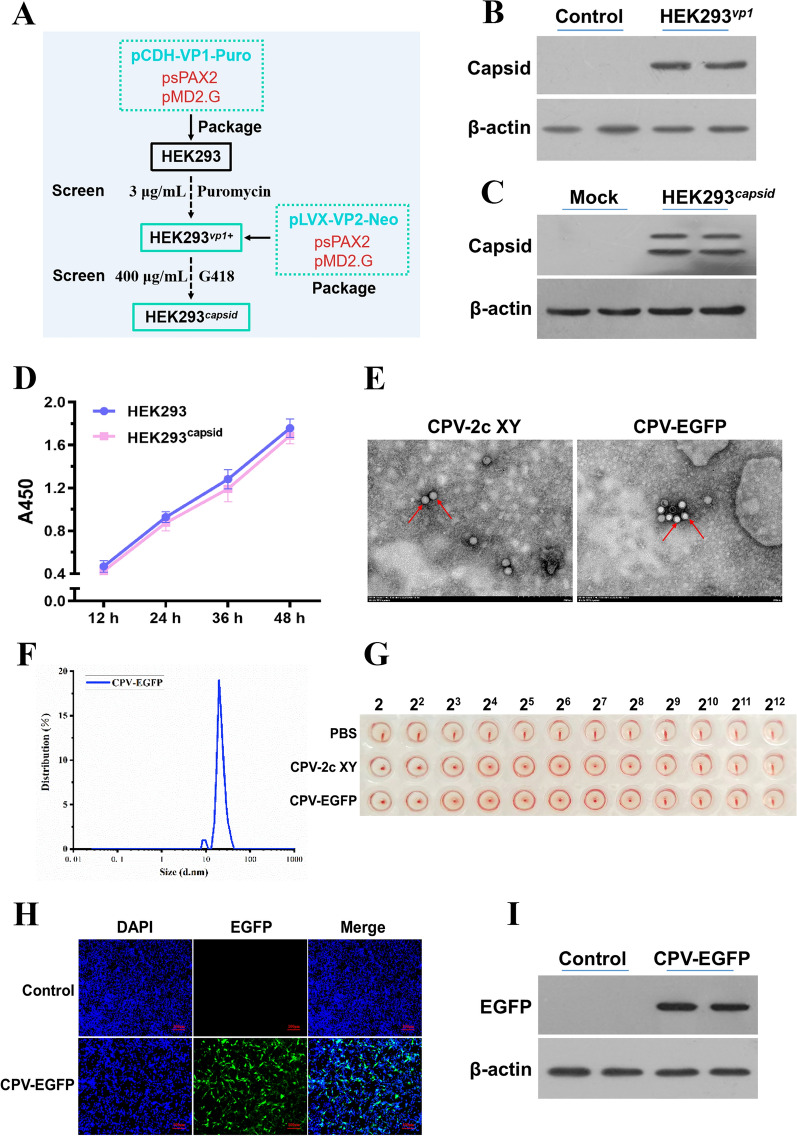
**Construction and identification of HEK293capsid cell line for CPV-2 pseudovirus assembly. A** The flowchart of screening the HEK293^*capsid*^ cell line. **B, C** Western blot analysis of the HEK293^*VP1*^ cell line **(B)** and the HEK293^*capsid*^ cell line **(C)**. **D** Growth curves of the HEK293^*capsid*^ cell line. **E** TEM analysis of CPV-EGFP assembly, scale bar = 200 nm. **F** The size distribution of CPV-EGFP was analyzed by DLS. **G** The characteristic of agglutinating pig erythrocytes of CPV-EGFP. **H, I** Immunofluorescence assay **(H)** and western blot analysis **(I)** of the expression of EGFP in CPV-EGFP infected F81 cells.

To construct a more convenient single-plasmid transfection system for the production of recombinant CPV-2 pseudoviruses, we generated a HEK293 cell line stably expressing CPV-2 capsids VP1 and VP2. The lentiviral plasmids expressing CPV-2 VP1 and VP2 were constructed with different antibiotic resistance genes as selectable markers (pCDH-VP1-puro and pLVX-VP2-Neo), respectively (Figure 3A). First, HEK293 cells expressing CPV-2 VP1 (HEK293^*VP1*^) were generated using pCDH-VP1-puro packaged lentivirus (Figure 3B). Subsequently, HEK293 cells expressing both CPV-2 VP1 and VP2 (HEK293^*capsid*^) were generated from HEK293^*VP1*^ using pLVX-VP2-Neo packaged lentivirus (Figure 3C). The HEK293^*capsid*^ cells had similar growth activity to wild-type HEK293 cells (Figure 3D). To figure out if the HEK293^*capsid*^ cells could be used to produce recombinant CPV-2 pseudovirus, we transfected the HEK293^*capsid*^ cells with the single exogenous EGFP gene transfer plasmid pCPV-EGFP. The results showed that the pCPV-EGFP transfected cells could also effectively produce recombinant CPV-2 pseudoviruses (CPV-EGFP), and the sizes of CPV-EGFP were also similar to wild-type CPV-2 (Figure 3E, 3F). The TCID_50_ of the recombinant CPV-2 pseudoviruses CPV-EGFP was 1 × 10^8.6^, and the agglutination capacity of CPV-EGFP was 1:2^8^ (Figure 3G). We infected F81 cells with the recombinant pseudovirus CPV-EGFP, and the infected cells also expressed high level of EGFP (Figure 3H, 3I). These results indicate that HEK293^*capsid*^ cells stably expressing CPV-2 VP1 and VP2 can be used to produce recombinant CPV-2 pseudovirus with the single transfection of the exogenous gene transfer plasmid.

### Packaging capacity of the recombinant CPV-2 pseudovirus for exogenous genes

**Figure 4:**
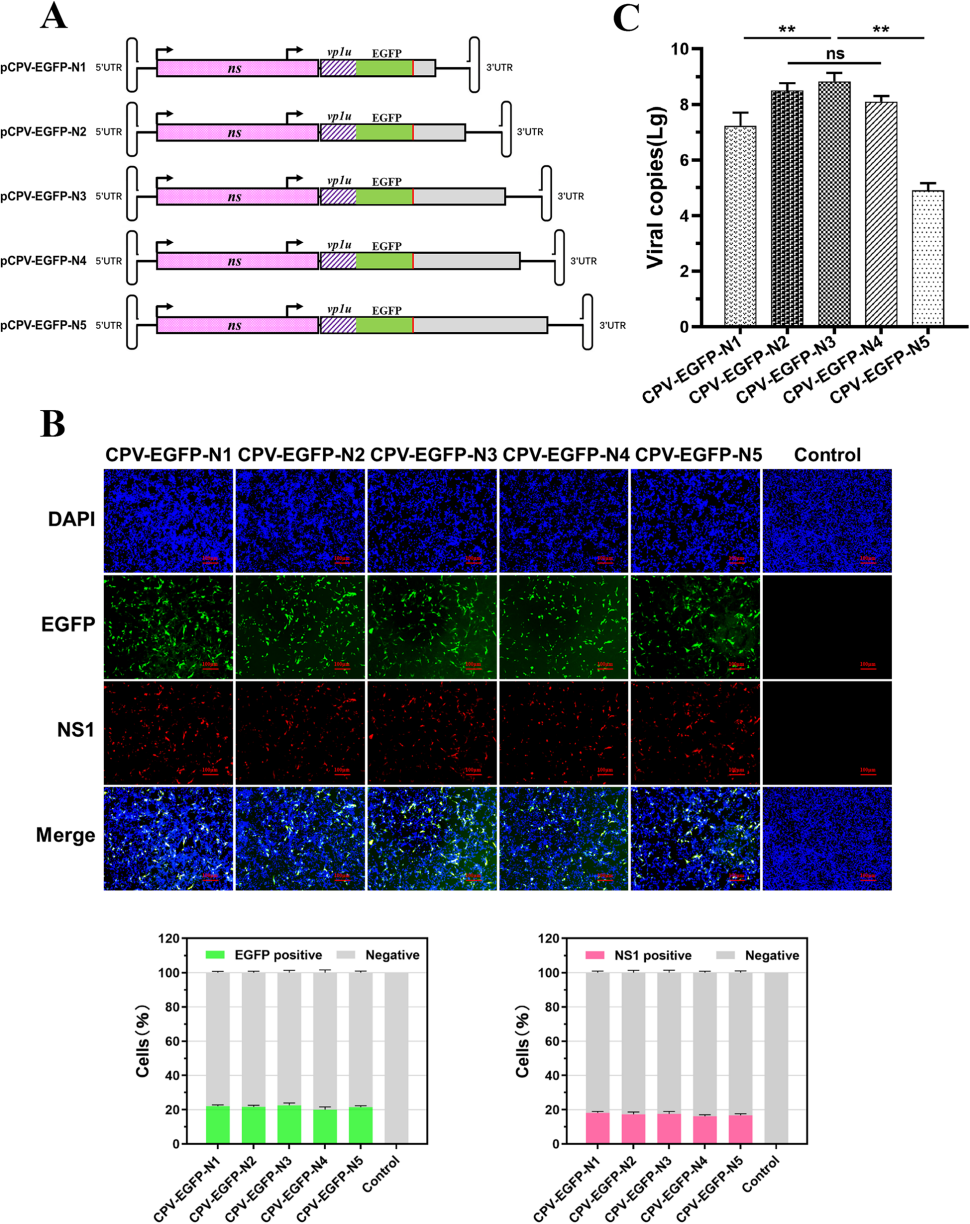
**The Capacity of recombinant CPV-2 pseudovirus. A** Schematic diagram of a series of CPV-2 pseudovirus transfer plasmids of different sizes. **B** Immunofluorescence assay of the expression of EGFP and NS1 in cells transfected with different lengths of the transfer plasmids. **C** The viral copies of recombinant CPV-2 pseudoviruses in cell culture supernatants.

To explore the packaging capacity of the recombinant CPV-2 pseudovirus, we constructed a series of exogenous gene transfer plasmids of different sizes by varying the length of the VP2 encoding region (57%, 80%, 100%, 120%, and 140% *vp2* with 999 bp, 1401 bp, 1755 bp, 2100 bp, and 2451 bp), ranging in size from 85 to 114% of CPV (85%, 93%, 100%, 107%, and 114% of CPV) (Figure 4A), and the sequence information is provided in Additional file 2. Then, we transfected these plasmids at the same amount into the HEK293^*capsid*^ cells stably expressing CPV-2 VP1 and VP2, respectively, and the results showed that all the transfected cells could express EGFP and CPV-2 NS1 in the same level (Figure 4B). We collected the supernatant of the transfected cells and detected the viral genome copies of the recombinant CPV-2 pseudoviruses by quantitative PCR, and the results showed that the copy numbers of the supernatant collected from the transfer plasmids containing 80% and 120% *vp2* transfected cells were at the same level of that in the plasmid containing 100% *vp2* transfected cell supernatant, while the copy numbers of the supernatant collected from the plasmids containing 57% and 140% *vp2* transfected cells were significantly lower (Figure 4C). These results show that the genome size allowing efficient packaging of recombinant CPV-2 pseudovirus ranges from 4600 to 5300 nt, and the insert size of the exogenous gene is in the range of 1400 bp to 2100 bp.

### Recombinant CPV-2 pseudovirus expressing CDV H protein induces efficient humoral immune responses against both CPV-2 and CDV

**Figure 5:**
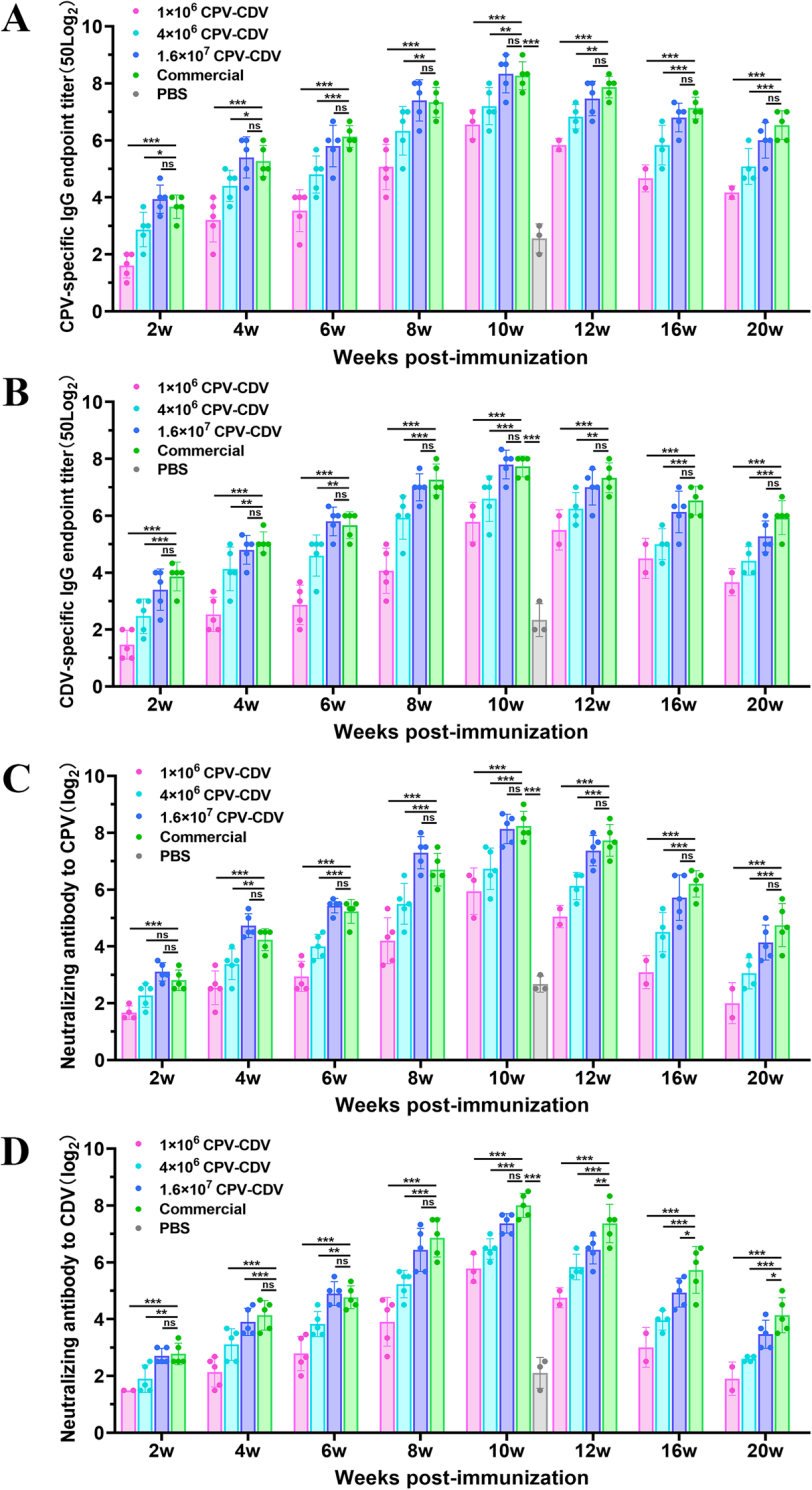
**Humoral immune responses induced by CPV–CDV in dogs.** Four groups of dogs (*n* = 5) were immunized with three different doses of CPV–CDV and the commercial vaccine, and the other group of dogs (*n* = 5) was immunized with PBS as the control. **A, B** Serum samples were collected for the detection of endpoint titers of total IgG against CPV-2 capsid proteins **(A)** and CDV H **(B)** by ELISA. **C, D** The virus neutralization test was performed to evaluate neutralization activity to CPV-2 **(C)** and CDV **(D)** of serum samples in each immunization group. The dots (•) of the corresponding colors in (**A, B**) and (**C, D**) represent endpoint titer and neutralization titer of individual dog, respectively. Statistical significance is indicated by asterisks (*): *p < 0.05, **p < 0.01, ***p < 0.001.

On the basis of the recombinant CPV-2 pseudovirus system, we produced a recombinant CPV-2 pseudovirus expressing hemagglutinin, the antigen of CDV. Basically, the gene of CDV H was subcloned into the transfer plasmid by replacing the VP2 coding region to construct the recombinant plasmid pCPV–CDV. Then the recombinant pCPV–CDV plasmid was transfected into HEK293^*capsid*^ cells to produce the recombinant pseudovirus CPV–CDV expressing CDV H protein. The recombinant pseudovirus CPV–CDV was collected and concentrated to 1 × 10^9^ TCID_50_ by ultracentrifugation. A total of 25 dogs were randomly divided into five groups, three groups were immunized with 1 × 10^6^ TCID_50_, 4 × 10^6^ TCID_50_, and 1.6 × 10^7^ TCID_50_ CPV–CDV pseudoviruses by intramuscular injection, respectively, one group was immunized with a commercial vaccine containing both live attenuated CPV-2 and CDV (containing 1 × 10^5^ TCID_50_ CPV-2 and 1 × 10^7^ TCID_50_ CDV), and the last one group was injected with the same volume of PBS. All groups of dogs were immunized three times at 0, 3, and 6 weeks post-immunization (wpi), and then challenged with 1 × 10^6^ TCID_50_ CPV-2 and 1 × 10^7^ TCID_50_ CDV at 8 wpi. The sera of the dogs were collected at 2, 4, 6, 8, 10, 12, 16, and 20 wpi, and the specific antibody level and neutralizing antibody level results showed that the different doses of CPV–CDV and the commercial vaccine immunization could gradually induce high levels of specific antibody and neutralizing antibody against both CPV-2 and CDV from 2 to 8 wpi, and 1.6 × 10^7^ TCID_50_ CPV–CDV pseudoviruses induced the same level of specific antibody and neutralizing antibody as the commercial vaccine (*p* > 0.05), whereas both 1 × 10^6^ TCID_50_ and 4 × 10^6^ TCID_50_ of CPV–CDV pseudoviruses induced lower antibody levels, starting from 2 wpi (*p* < 0.05) (Figure 5A–D). The specific antibody and neutralizing antibody levels reached peak at 10 wpi (2 week post-challenge with CPV-2 and CDV) and then decreased (Figure 5A–D). These results demonstrate that at a dose of 1.6 × 10^7^ TCID_50_, the recombinant CPV-2 pseudovirus expressing CDV H protein could induce the similar level of specific antibody and neutralizing antibody as the commercial live attenuated vaccine to against both CPV-2 and CDV.

### Recombinant CPV-2 pseudovirus expressing CDV H protein elicits T cell activation, accompanied by the serological characteristic of a higher IgG2a/IgG1 ratio

**Figure 6:**
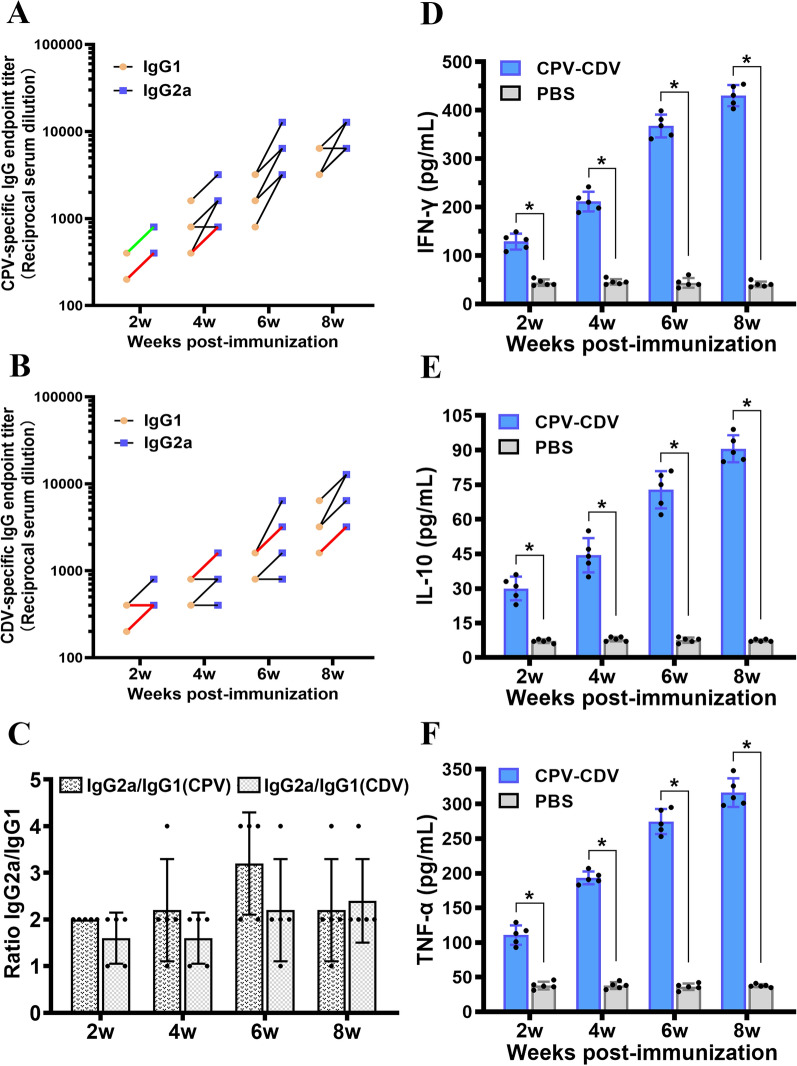
**Analysis of IgG subclass profiles and cytokine responses. A, B** The IgG2a and IgG1 endpoint titers against CPV-2 capsid proteins **(A)** and CDV H **(B)** of the highest dose group (1.6 × 10^7^ TCID_50_). **C** The IgG2/IgG1 ratio against CPV-2 capsid proteins and CDV H. Black dots (•) represent IgG2/IgG1 ratio of individual dog. The red lines indicate the endpoint titers of two dogs with the same value. The green lines indicate the endpoint titers of three dogs with the same value. **D–F** The detection of IFN-γ **(D)**, TNF-α **(E)**, and IL-10 **(F)** in serum samples from the highest dose group (1.6 × 10^7^ TCID_50_). Black dots (•) represent cytokines level of individual dog. Asterisk (*) indicates significant difference (*p* < 0.05).

T cell activation is crucial for vaccine-induced immunity. To further evaluate the immune response elicited by the recombinant pseudovirus CPV–CDV, we analyzed the profiles of antigen-specific IgG subclasses and related cytokines. Levels of IgG1 and IgG2a against CPV-2 and CDV in sera were detected prior to challenge. The results showed that after immunization, IgG2a levels in each dog were higher than or at least equal to IgG1 levels, and both IgG2a and IgG1 levels increased from 2 to 8 wpi (Figure 6A, B). In addition, the IgG2a/IgG1 ratios against CPV-2 and CDV were relatively stable at all time points (Figure 6C). We further examined the secretion profiles of cytokines involved in cell-mediated immunity or immunomodulatory functions. The levels of IFN-γ, TNF-α, and IL-10 gradually increased over time after immunization (Figure 6D–F). Collectively, the elevated antigen-specific IgG subclasses and enhanced cytokine secretion from immune-related cells demonstrated that the recombinant pseudovirus CPV–CDV effectively induced T helper cell activation and functional cellular immune responses.

### The sera from recombinant CPV-2 pseudovirus expressing CDV H protein immunized dogs recognize multiple linear B cell epitopes and CD8^+^ T Cell immunity associated epitopes of both CPV-2 and CDV

**Figure 7:**
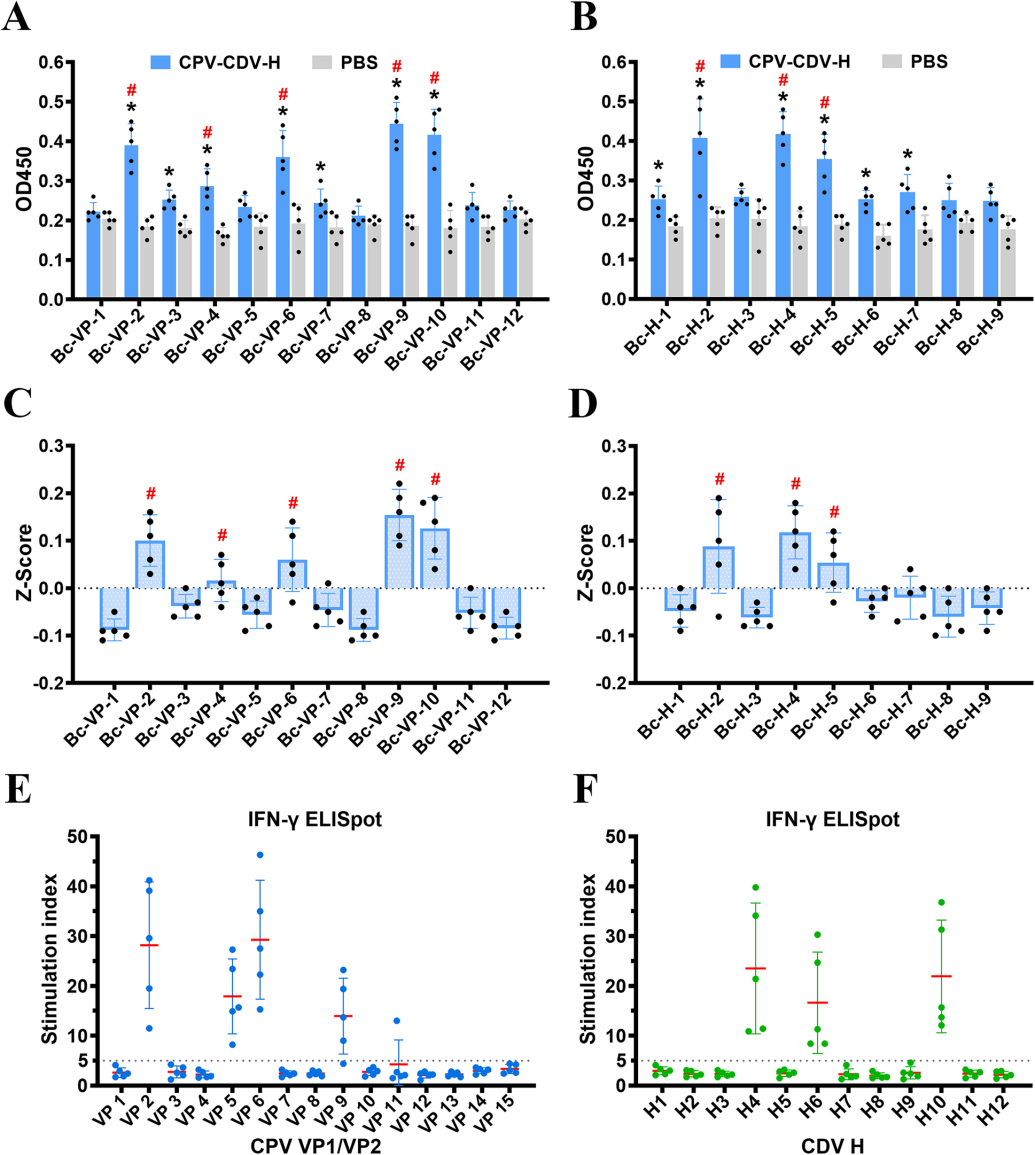
**Responses against predicted linear B cell and T cell epitopes. A–D** IgG response to each predicted linear B-cell epitope of CPV-2 capsid protein **(A)** and each predicted linear B-cell epitope of CDV H **(B)**. Determination of immunodominant epitopes of CPV-2 capsid protein **(C)** and CDV H **(D)** based on Z-score. Black dot (•) represents value of individual dog. Serum samples immunized with CDV-H and the PBS control group were tested with synthetic peptides. A peptide showing Z-score mean > 0.04 (cutoff indicated by dashed line (–)) was considered immunodominant. Asterisk (*) indicates a significant difference between CDV H group and PBS control group (*p* < 0.05). Octothorpe (#) indicates immunodominant epitope. **E, F** The detection of IFN-γ secreting cells was performed by re-stimulating the splenocytes with peptide pools. **E** The T cell response to peptide pools of CPV-2 capsid proteins. **F** The T cell response to peptide pools of CDV H.

To further analyze the ability of sera antibodies produced in CPV–CDV-immunized dogs to recognize CPV-2 and CDV antigens, we synthesized a series of peptides corresponding to predicted linear B cell epitopes (BepiPred-2.0) of CPV-2 VP1/VP2 (named as Bc-VP-1 to Bc-VP-12) and CDV H (named as Bc-H-1 to Bc-H-9) (Table 1), and these epitope peptides were used to detect the reactivity with the immunized sera from the dogs at 8 wpi. The results showed that the sera of CPV–CDV immunized dogs could effectively react with seven peptides of CPV-2 VP1/VP2 (Bc-VP-2, Bc-VP-3, Bc-VP-4, Bc-VP-6, Bc-VP-7, Bc-VP-9, and Bc-VP-10) and six peptides of CDV H (Bc-H-1, Bc-H-2, Bc-H-4, Bc-H-5, Bc-H-6, and Bc-H-7) comparing with the sera of PBS group dogs (Figure 7A, B). On the basis of Z-score analysis results, five peptides of CPV-2 VP1/VP2 (Bc-VP-2, Bc-VP-4, Bc-VP-6, Bc-VP-9, and Bc-VP-10) and three peptides of CDV H (Bc-H-2, Bc-H-4, and Bc-H-5) were recognized to be the most immunogenic antigen peptides (Figure 7C, 7D). Besides, we predicted and synthesized peptides corresponding to CD8^+^ T cell epitopes of CPV-2 VP1/VP2 (designated VP1-VP15) and CDV H (designated H1-H12) based on dog MHC alleles (dog leukocyte antigen, DLA) using NetMHCpan EL 4.1 and NetCTL 1.2 Server (Table 2). Then the splenocytes from CPV–CDV immunized dogs were stimulated with these epitope peptides, and the IFN-γ secretion levels were measured. The results showed that the peptides VP2, VP5, VP6, and VP9 induced higher levels of IFN-γ secretion level than other peptides of CPV-2 VP1/VP2, while the peptides H4, H6, and H10 induced higher levels of IFN-γ secretion level than other peptides of CDV H (Figure 7E, 7F). These results show that the immunization of recombinant CPV-2 pseudovirus expressing CDV H protein in dogs could induce secretion of specific antibodies and activation of cytotoxic T cell response.

**Table 1:** **Predicted B cell epitopes of the CPV VP2 and CDV H proteins**

Source	Peptide Name	Peptide	Start	End	Length
CPV-2 VP1/VP2	VP 1	PGNSLDQGEPTNPSD	22	36	15
	VP 2	TSRPSKPTKRSKPP	105	118	14
	VP 3	NLAPMSDGGVQ	140	150	11
	VP 4	AVRNERATGSGNGSG	157	171	15
	VP 5	NNQTEFKFLENG	189	200	12
	VP 6	VNNLDKTAVNGNMALDD	227	243	17
	VP 7	TLIPSHTGTSGTPTNIYHG	360	378	19
	VP 8	AEGGTNFGYIGVQQDKRRGVTQMG	440	463	24
	VP 9	GGAQTDENQAADGD	505	518	14
	VP 10	QKTTTTGETPER	529	540	12
	VP 11	LTNEYDPDASANM	649	661	13
	VP 12	WNPIQQMSINVDNQFNYV	688	705	18
CDV H	H 1	GAFYKDNARANS	9	20	12
	H 2	VTEEQGGRRP	26	35	10
	H 3	LKEDMEKSEAVHHQV	74	88	15
	H 4	LSALSGGRGDIFPPYRCSG	172	190	19
	H 5	YHDSNGSQDGI	305	315	11
	H 6	VSEKQEEQKNCLES	367	380	14
	H 7	GMDYYESPLLDSG	436	448	13
	H 8	NKASRGDQFTVIPHV	464	478	15
	H 9	ADITNSTTSVENLVRIR	583	599	17

**Table 2:** **Predicted CD8 + T cell immunity associated epitopes**

Source	Peptide name	Peptide	Start	End	Length	Tool	Allele
CPV-2 VP1/VP2	VP 1	LSYQDKVSAFY	2	12	11	NetMHCpan	DLA-8850101, DLA-8803401, DLA-8850801
	VP 2	QVSTSNMEF	62	70	9	NetCTL	
	VP 3	QVIDVLTPLF	87	96	10	NetMHCpan	
	VP 4	KLNEIKQFIL	110	119	10	NetMHCpan	
	VP 5	LTAISDGVY	220	228	9	NetCTL	
	VP 6	KTYLLVHDY	230	238	9	NetCTL	
	VP 7	RVFEIGFIKR	249	258	10	NetMHCpan	
	VP 8	CVDESTVLLY	296	305	10	NetCTL	
	VP 9	KIHITNHRGF	344	353	10	NetMHCpan	
	VP 10	ESACQRKSY	379	387	9	NetCTL	
	VP 11	ILNGDGMDY	431	439	9	NetCTL	
	VP 12	LLDSGWLTI	444	452	9	NetCTL	
	VP 13	STVIPHVFTF	472	481	10	NetMHCpan	
	VP 14	VVLPTQNFRY	511	520	10	NetMHCpan	
	VP 15	YTYPFRLTTK	547	556	10	NetMHCpan	
CDV H	H 1	RGLVPPGYKY	10	19	10	NetMHCpan	DLA-8850101, DLA-8803401, DLA-8850801
	H 2	YLRSGKNPYLY	49	59	11	NetCTL	
	H 3	KRSKPPPHIF	113	122	10	NetMHCpan	
	H 4	LVDANAWGV	254	262	9	NetCTL	
	H 5	TMSELHLVSF	275	284	10	NetMHCpan	
	H 6	ATQPPTKVY	300	308	9	NetCTL	
	H 7	AAMRSETLGFY	331	341	11	NetMHCpan	
	H 8	KPTIPTPWRY	344	353	10	NetMHCpan	
	H 9	TSGTPTNIY	368	376	9	NetCTL	
	H 10	GTDPDDVQF	378	386	9	NetCTL	
	H 11	QAADGDPRY	513	521	9	NetCTL	
	H 12	IAHQDTGRY	544	552	9	NetCTL	

The prediction was performed with the dog MHC allele (dog leukocyte antigen, DLA)

### The recombinant CPV-2 pseudovirus expressing CDV H protein immunization protects dogs from the challenge of both CPV-2 and CDV

**Figure 8:**
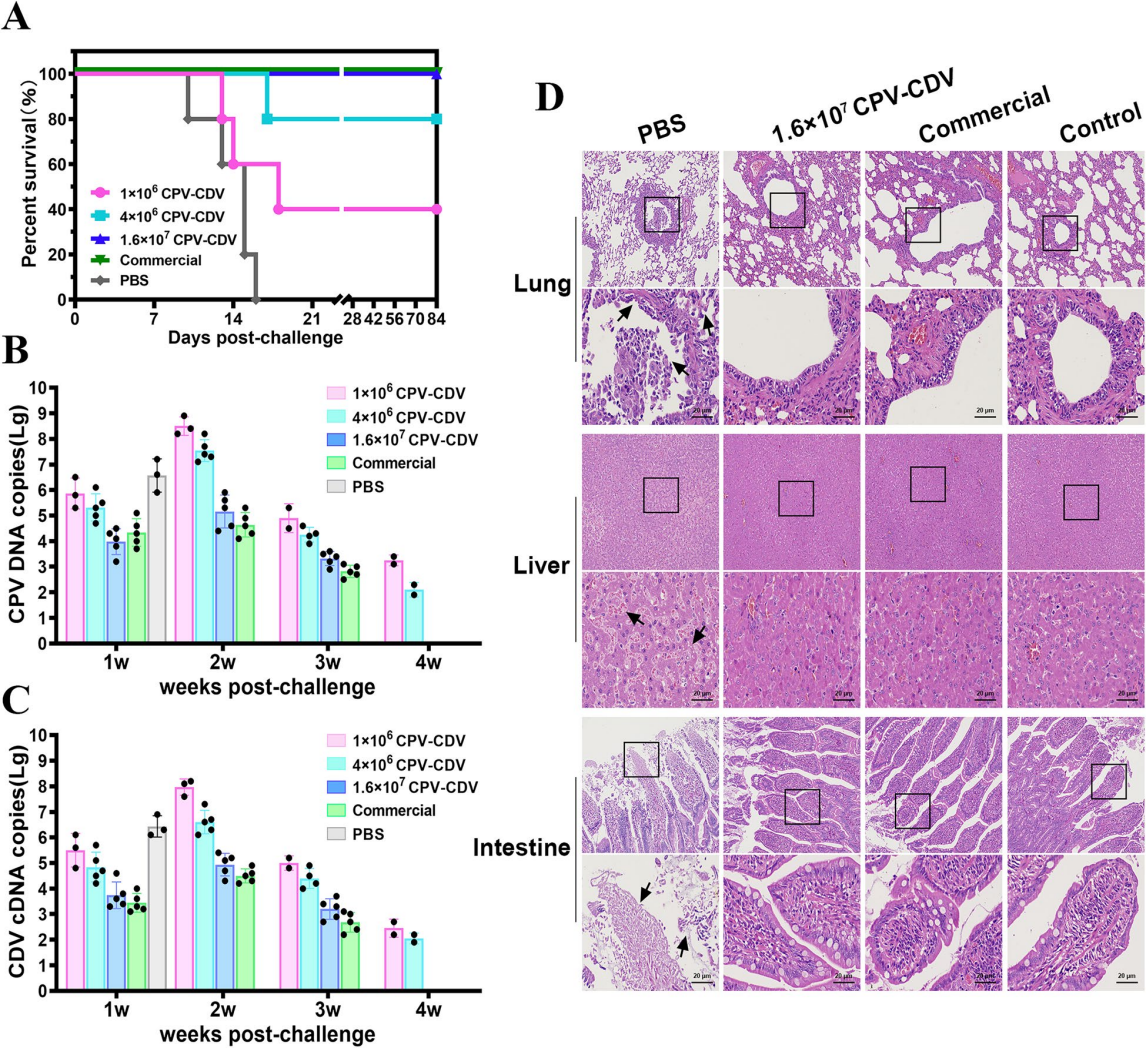
**CPV–CDV effectively protects dogs from CPV-2 and CDV infectious challenges. A** The survival rates of the CPV-2 and CDV challenged dogs in different groups. **B, C** The CPV-2 DNA copy numbers **(B)** and CDV RNA copy numbers **(C)** in fecal samples from 1 to 4 weeks after challenge were detected by RT-qPCR. Samples are considered as negative when CPV-2 DNA loads/g or CDV RNA loads/g are less than 10^2^. **D** The histopathological features of lungs, liver, and intestine. Black arrows ( →) indicate the typical pathological changes of each tissue respectively: in the lung tissue, the arrows indicate the disordered alveolar structure, degeneration, necrosis, and detachment of alveolar epithelial cells, widened pulmonary interstitium, inflammatory cell infiltration; in the liver tissue, the arrows indicate hepatocyte degeneration and necrosis, dilation, and congestion of hepatic sinusoids, inflammatory cell infiltration; in the intestinal tissue, the arrows indicate the degeneration, necrosis, and shedding of intestinal mucosal epithelial cells, inflammatory cell infiltration in the lamina propria, atrophy, and rupture of intestinal villi, and disordered glandular architecture. Scale bar = 20 μm.

The dogs immunized with CPV–CDV, commercial vaccine, and PBS were challenged with CPV-2 or CDV at 8 wpi. The results showed that all the dogs immunized with PBS had died by 16 days post-challenge (dpc), whereas two of five dogs immunized with 1 × 10^6^ TCID_50_ CPV–CDV pseudoviruses had survived at 84 dpc, four of five dogs immunized with 4 × 10^6^ TCID_50_ CPV–CDV pseudoviruses had survived at 84 dpc, and all the dogs immunized with 1.6 × 10^7^ TCID_50_ CPV–CDV pseudoviruses had survived at 84 dpc (Figure 8A). The CPV-2 DNA and CDV cDNA were detected per week from the feces by quantitative RT-PCR, and the results showed that the survived PBS group dogs had a higher both CPV-2 and CDV loads in the feces than the immunization group dogs at 1 week post-challenge (wpc), then none viruses were detected since all the PBS group dogs died (Figure 8B, C). The viral loads of the different doses of CPV–CDV and commercial vaccine group dogs were all gradually decreased, and the viral loads of the 1.6 × 10^7^ TCID_50_ CPV–CDV pseudoviruses immunized dogs and the commercial vaccine immunized dogs were lower than that of the 1 × 10^6^ TCID_50_ CPV–CDV pseudoviruses immunized dogs and the 4 × 10^6^ TCID_50_ CPV–CDV pseudoviruses immunized dogs at all time points, and even undetectable at 4 wpc (Figure 8B, C). All the challenged dogs in the PBS group presented severe clinical symptoms, with breathing difficulties, hemorrhagic diarrhea, and neurological symptoms. The challenged PBS group dogs showed detachment and necrosis of bronchial airway epithelial cells and vacuolization of epithelial cells with cytoplasmic and cytosolic nuclei containing a large number of viral inclusion bodies, accompanied by macrophage hyperplasia in lungs, cellular vacuolar degeneration, and necrosis as well as damage to the cell cord structure in livers, and necrosis and shedding of intestinal epithelial cells in intestines (Figure 8D). By contrast, the tissues of the 1.6 × 10^7^ TCID_50_ CPV–CDV pseudoviruses immunized dogs and the commercial vaccine immunized dogs showed no significant changes compared with the nonchallenged control dogs (Figure 8D). These results confirm that the vaccination with the recombinant CPV–CDV pseudovirus could protect dogs against the challenge of CPV-2 and CDV as the commercial live vaccine.

## Discussion

Replication-deficient pseudoviruses are capable of infecting host cells and are considered natural nanocarriers for effective transmission of genetic material when used as vaccine vectors [29, 30]. At present, some viruses have been engineered into replication-deficient pseudovirus vectors to develop vaccines or therapeutics, such as adenovirus (AdV), AAV, vesicular stomatitis virus (VSV), Newcastle disease virus (NDV), and so on [18, 30–33]. The advantage of developing vectors of the family *Parvoviridae* as gene delivery systems lies in their simple genetic structure, which facilitates the modification and engineering of their genomes and capsid proteins. This allows not only the precise insertion of exogenous sequences, but also the directional modification of viral capsids [24, 25, 34, 35]. In addition to AAV, the APVs have also been reported to be adept at mediating the transient and efficient expression of exogenous genes [19, 21, 35]. In the present study, we engineered CPV-2 to be a replication-deficient pseudovirus vector, and developed a CPV–CDV pseudovirus expressing CDV H protein that could protect dogs from both CPV-2 and CDV infections with high safety.

We previously successfully constructed the reverse genetic system of CPV-2 to realize the manipulation of the virus at gene level [28]. The structural proteins VP1 and VP2 are the main subunit components of parvovirus virions and exhibit good immunogenicity because they contain numerous crucial antigenic epitopes, thereby inducing effective neutralizing antibodies [36]. VP1 and VP2 are encoded by the positive-sense genomic DNA, and the VP1 coding sequence contains the VP2 coding sequence, which shares the same C-terminus, meaning that deletion of the VP2 coding sequence would result in the failure of the expression of both VP1 and VP2 [37]. However, owing to the limited genome size of CPV-2, the gene fragments of the virus are highly overlapping and the gene expression regulatory elements are compact, which make it difficult to delete all the coding sequences of VP1 and VP2 [38]. So, in this study, we constructed the replication-deficient CPV-2 pseudovirus vector by replacing the VP2 coding sequence to the exogenous gene sequences. Meanwhile, we retained the complete sequence of *ns1*, as previous studies have demonstrated that the C-terminus of the NS1 coding sequence exerts a regulatory role in the transcription of structural proteins, and NS1 protein may be involved in immunostimulatory effects [17, 39]. However, it should be noted that this construction strategy limits the capacity of the pseudovirus so that it can only insert foreign sequences with a size of 1400–2100 nt. Our research found that when the genome sequence length exceeds 2100 nt, although it does not affect the gene expression, it significantly reduces the packaging efficiency of the recombinant pseudoviruses, which is consistent with previous studies on other APVs [40, 41]. On the basis of the further knowledge about the genome structure of CPV-2, better strategies would be explored in the future to expand the insertable capacity. Furthermore, because of the replacement of VP2 coding sequence, the viral structural proteins are needed to generate viral particles. In previous studies, the two-component VP1 and VP2 capsid was found to have biological properties more similar to natural virus than the self-assembled VP2 capsid [42]. Thus, we generated a HEK293 cell line that stably expresses CPV-2 VP1 and VP2 using lentivirus system for the efficient production of pseudovirus, yet a more suitable cell line is still necessary to reduce the production cost of recombinant pseudoviruses and improve production efficiency and stability.

It is noteworthy that the potential recombination risk between recombinant viral vectors and wild-type viruses is a key biosafety concern [43, 44]. In the context of our CPV-2 pseudovirus platform, key strategies were designed to eliminate this risk of recombination with wild-type live CPV. First, the stable cell line HEK293^*capsid*^ is utilized to provide CPV-2 VP1 and VP2, and only a single recombinant plasmid is transfected to produce recombinant pseudoviruses, completely removing homologous arms of both the transfer plasmid and the plasmids providing VP1/VP2 proteins, thus avoiding the prerequisite for homologous recombination. Second, the recombinant CPV-2 pseudoviruses produced in this system are replication-defective and unable to generate progeny infectious viruses; they can effectively express the target antigen while completely losing their replication capacity, thus preventing the generation of novel recombinant viruses in vivo. In addition, we continuously performed sequencing of the product to monitor potential recombination events throughout the virus preparation and animal experiments.

Pseudovirus vectors expressing chimeric antigens have emerged as important tools in modern vaccine research, and facilitate the design of multivalent vaccines by incorporating antigens from multiple pathogens or serotypes [45, 46]. These vaccines elicit robust immune responses while maintaining safety profiles [46]. In this study, we evaluated the protective efficacy and immune responses induced in dogs by a recombinant CPV-2 pseudovirus expressing CDV H protein (CPV–CDV) against challenge with both CPV-2 and CDV. The high dose of recombinant pseudovirus CPV–CDV was found to stimulate high levels of CPV-2 and CDV-specific antibodies and neutralizing antibodies, and CPV–CDV was shown to be sufficient to confer complete protection against both CPV-2 and CDV infections. T cell activation is critical for antiviral and vaccine-induced immune responses [47, 48], particularly the differentiation of CD4 + T cells that support B cell clonal expansion and antibody affinity maturation [49]. Both CPV-specific and CDV-specific antibodies were predominantly of the IgG2a isotype. Although the ratio of IgG2 to IgG1 has served as an indicator to evaluate Th1/Th2 polarization in some previous studies on canine vaccines [50], the complex regulatory mechanisms underlying Th1/Th2 polarization and the corresponding cytokine secretion in dogs have not been fully determined, and there is still a lack of theoretical basis and experimental verification. In addition, the secretion levels of IFN-γ, IL-10, and TNF-α were all significantly increased after immunization, which contributed to antiviral activity. Taking into account the good immune effect of the recombinant pseudovirus CPV–CDV in dogs, we investigated its immunogenicity characteristics. A number of dominant linear B-cell epitopes located in the CPV-2 capsid protein and CDV H protein were determined. It is also worth noting that the developing vaccines using whole capsid proteins as immunogens could provide better immune induction [51]. The capsid proteins of recombinant pseudoviruses maintain their tertiary structures and possess conformational epitopes being difficult to identify, which may induce higher levels of antibody response, compared with multi-epitope immunogens composed of linear epitopes. In terms of T cell response, the regions containing potential immunodominant T-cell epitopes were preliminarily identified, which has reference significance for the development of vaccines targeting CPV-2 VP2 protein and CDV H protein with high mutation frequency. Furthermore, on the basis of the established immunogenicity profile of our novel pseudovirus vaccine, it would be valuable to compare its epitope specificity with that induced by commercial vaccines or natural infections in future studies. This will help evaluate and potentially optimize the ability of different vaccine candidates to direct the immune response toward the most protective and conserved antigenic sites.

In conclusion, we have successfully generated a recombinant pseudovirus system based on CPV-2 genome. The produced recombinant pseudovirus CPV–CDV showed preferable humoral and cellular immunity in dogs. These results indicate the potential of recombinant CPV-2 pseudoviruses as vaccine candidates and lay the foundation for developing them as novel vaccines.

## Supplementary Information

**Additional file 1** **Sequences of insert genes.**

**Additional file 2** **Animal Welfare and Ethical Safeguards.**

**Additional file 3** **Predicted B cell epitopes of the CPV VP2 and CDV H proteins.**

**Additional file 4** **Predicted CD8+ T cell immunity associated epitopes of CPV-2 VP1/VP2 and CDV H using NetMHCpan EL 4.1 and NetCTL 1.2 Server.** The prediction was performed with the dog MHC allele (dog leukocyte antigen, DLA).

## Data Availability

No datasets were generated or analyzed during the current study.
