# Supplementary material for: Development of canine parvovirus-2-based recombinant pseudoviruses expression system: a potential vaccine platform
Source: Vet Res. 2026 Jun 18;57:110. doi: 10.1186/s13567-026-01789-9 (PMC13277292; doi:10.1186/s13567-026-01789-9)
Supplement: Supplementary file 1 — Additional file 1 Sequences of insert genes. [file 13567_2026_1789_MOESM1_ESM.docx]

**Additional File 1**

**The insertion sequence of 999bp：**

**ATGGTGAGCAAGGGCGAGGAGCTGTTCACCGGGGTGGTGCCCATCCTGGTCGAGCTGGACGGCGACGTAAACGGCCACAAGTTCAGCGTGTCCGGCGAGGGCGAGGGCGATGCCACCTACGGCAAGCTGACCCTGAAGTTCATCTGCACCACCGGCAAGCTGCCCGTGCCCTGGCCCACCCTCGTGACCACCCTGACCTACGGCGTGCAGTGCTTCAGCCGCTACCCCGACCACATGAAGCAGCACGACTTCTTCAAGTCCGCCATGCCCGAAGGCTACGTCCAGGAGCGCACCATCTTCTTCAAGGACGACGGCAACTACAAGACCCGCGCCGAGGTGAAGTTCGAGGGCGACACCCTGGTGAACCGCATCGAGCTGAAGGGCATCGACTTCAAGGAGGACGGCAACATCCTGGGGCACAAGCTGGAGTACAACTACAACAGCCACAACGTCTATATCATGGCCGACAAGCAGAAGAACGGCATCAAGGTGAACTTCAAGATCCGCCACAACATCGAGGACGGCAGCGTGCAGCTCGCCGACCACTACCAGCAGAACACCCCCATCGGCGACGGCCCCGTGCTGCTGCCCGACAACCACTACCTGAGCACCCAGTCCGCCCTGAGCAAAGACCCCAACGAGAAGCGCGATCACATGGTCCTGCTGGAGTTCGTGACCGCCGCCGGGATCACTCTCGGCATGGACGAGCTGTACAAGGGTGGCGGTGGAAGTATCGACTGCCGATACGATCGATACCGAGATTCTCTGAGGACCGTGGGCTTTCTGCTACATCTATCCATCGGTCGTAGGATCCATGATCGATACATCAATCGCTCGAATCACAGCGTCGCACTACACGAACTAAATGTACTTGAGATACGTCTATGTGTGCGCCAGAAGTGCCTTGTACTAAGCTGTTCGATACGAACCATCGATCGTTCTACGATCGATTGGTCATACAATCGATCGCTACATGAGCTTGTCAATTCACTCGACTGA**

**The insertion sequence of 1401bp：**

**ATGGTGAGCAAGGGCGAGGAGCTGTTCACCGGGGTGGTGCCCATCCTGGTCGAGCTGGACGGCGACGTAAACGGCCACAAGTTCAGCGTGTCCGGCGAGGGCGAGGGCGATGCCACCTACGGCAAGCTGACCCTGAAGTTCATCTGCACCACCGGCAAGCTGCCCGTGCCCTGGCCCACCCTCGTGACCACCCTGACCTACGGCGTGCAGTGCTTCAGCCGCTACCCCGACCACATGAAGCAGCACGACTTCTTCAAGTCCGCCATGCCCGAAGGCTACGTCCAGGAGCGCACCATCTTCTTCAAGGACGACGGCAACTACAAGACCCGCGCCGAGGTGAAGTTCGAGGGCGACACCCTGGTGAACCGCATCGAGCTGAAGGGCATCGACTTCAAGGAGGACGGCAACATCCTGGGGCACAAGCTGGAGTACAACTACAACAGCCACAACGTCTATATCATGGCCGACAAGCAGAAGAACGGCATCAAGGTGAACTTCAAGATCCGCCACAACATCGAGGACGGCAGCGTGCAGCTCGCCGACCACTACCAGCAGAACACCCCCATCGGCGACGGCCCCGTGCTGCTGCCCGACAACCACTACCTGAGCACCCAGTCCGCCCTGAGCAAAGACCCCAACGAGAAGCGCGATCACATGGTCCTGCTGGAGTTCGTGACCGCCGCCGGGATCACTCTCGGCATGGACGAGCTGTACAAGGGTGGCGGTGGAAGTATCGACTGCCGATACGATCGATACCGAGATTCTCTGAGGACCGTGGGCTTTCTGCTACATCTATCCATCGGTCGTAGGATCCATGATCGATACATCAATCGCTCGAATCACAGCGTCGCACTACACGAACTAAATGTACTTGAGATACGTCTATGTGTGCGCCAGAAGTGCCTTGTACTAAGCTGTTCGATACGAACCATCGATCGTTCTACGATCGATTGGTCATACAATCGATCGCTACATGAGCTTGTCAATTCACTCGACGGTGGAGGTGGAAGTGGTGGAGGTGGAAGTGGTGGAGGTGGAAGTGGTGGAGGTGGAAGTGGTGGAGGTGGAAGTGGTGGAGGTGGAAGTGGTGGAGGTGGAAGTGGTGGAGGTGGAAGTGGTGGTTCTGGTGGTAGTATCGACTGCCGATACGATCGATACCGAGATTCTCTGAGGACCGTGGGCTTTCTGCTACATCTATCCATCGGTCGTAGGATCCATGATCGATACATCAATCGCTCGAATCACAGCGTCGCACTACACGAACTAAATGTACTTGAGATACGTCTATGTGTGCGCCAGAAGTGCCTTGTACTAAGCTGTTCGATACGAACCATCGATCGTTCTACGATCGATTGGTCATACAATCGATCGCTACATGAGCTTGTCAATTCACTCGAC TGA**

**The insertion sequence of 1755bp：**

**ATGGTGAGCAAGGGCGAGGAGCTGTTCACCGGGGTGGTGCCCATCCTGGTCGAGCTGGACGGCGACGTAAACGGCCACAAGTTCAGCGTGTCCGGCGAGGGCGAGGGCGATGCCACCTACGGCAAGCTGACCCTGAAGTTCATCTGCACCACCGGCAAGCTGCCCGTGCCCTGGCCCACCCTCGTGACCACCCTGACCTACGGCGTGCAGTGCTTCAGCCGCTACCCCGACCACATGAAGCAGCACGACTTCTTCAAGTCCGCCATGCCCGAAGGCTACGTCCAGGAGCGCACCATCTTCTTCAAGGACGACGGCAACTACAAGACCCGCGCCGAGGTGAAGTTCGAGGGCGACACCCTGGTGAACCGCATCGAGCTGAAGGGCATCGACTTCAAGGAGGACGGCAACATCCTGGGGCACAAGCTGGAGTACAACTACAACAGCCACAACGTCTATATCATGGCCGACAAGCAGAAGAACGGCATCAAGGTGAACTTCAAGATCCGCCACAACATCGAGGACGGCAGCGTGCAGCTCGCCGACCACTACCAGCAGAACACCCCCATCGGCGACGGCCCCGTGCTGCTGCCCGACAACCACTACCTGAGCACCCAGTCCGCCCTGAGCAAAGACCCCAACGAGAAGCGCGATCACATGGTCCTGCTGGAGTTCGTGACCGCCGCCGGGATCACTCTCGGCATGGACGAGCTGTACAAGGGTGGCGGTGGAAGTATCGACTGCCGATACGATCGATACCGAGATTCTCTGAGGACCGTGGGCTTTCTGCTACATCTATCCATCGGTCGTAGGATCCATGATCGATACATCAATCGCTCGAATCACAGCGTCGCACTACACGAACTAAATGTACTTGAGATACGTCTATGTGTGCGCCAGAAGTGCCTTGTACTAAGCTGTTCGATACGAACCATCGATCGTTCTACGATCGATTGGTCATACAATCGATCGCTACATGAGCTTGTCAATTCACTCGACGGTGGAGGTGGAAGTGGTGGAGGTGGAAGTGGTGGAGGTGGAAGTGGTGGAGGTGGAAGTGGTGGAGGTGGAAGTGGTGGAGGTGGAAGTGGTGGAGGTGGAAGTGGTGGAAGTATCGACTGCCGATACGATCGATACCGAGATTCTCTGAGGACCGTGGGCTTTCTGCTACATCTATCCATCGGTCGTAGGATCCATGATCGATACATCAATCGCTCGAATCACAGCGTCGCACTACACGAACTAAATGTACTTGAGATACGTCTATGTGTGCGCCAGAAGTGCCTTGTACTAAGCTGTTCGATACGAACCATCGATCGTTCTACGATCGATTGGTCATACAATCGATCGCTACATGAGCTTGTCAATTCACTCGACGGTGGAGGTGGAAGTGGTGGAGGTGGAAGTGGTGGAGGTGGAAGTGGTGGAGGTGGAAGTGGTGGAGGTGGAAGTGGTGGAGGTGGAAGTGGTGGAGGTGGAAGTGGTGGAAGTATCGACTGCCGATACGATCGATACCGAGATTCTCTGAGGACCGTGGGCTTTCTGCTACATCTATCCATCGGTCGTAGGATCCATGATCGATACATCAATCGCTCGAATCACAGCGTCGCACTACACGAACTAAATGTACTTGAGATACGTCTATGTGTGCGCCAGAAGTGCCTTGTACTAAGCTGTTCGATACGAACCATCGATCGTTCTACGATCGATTGGTCATACAATCGATCGCTACATGAGCTTGTCAATTCACTCGACTGA**

**The insertion sequence of 2100bp： ATGGTGAGCAAGGGCGAGGAGCTGTTCACCGGGGTGGTGCCCATCCTGGTCGAGCTGGACGGCGACGTAAACGGCCACAAGTTCAGCGTGTCCGGCGAGGGCGAGGGCGATGCCACCTACGGCAAGCTGACCCTGAAGTTCATCTGCACCACCGGCAAGCTGCCCGTGCCCTGGCCCACCCTCGTGACCACCCTGACCTACGGCGTGCAGTGCTTCAGCCGCTACCCCGACCACATGAAGCAGCACGACTTCTTCAAGTCCGCCATGCCCGAAGGCTACGTCCAGGAGCGCACCATCTTCTTCAAGGACGACGGCAACTACAAGACCCGCGCCGAGGTGAAGTTCGAGGGCGACACCCTGGTGAACCGCATCGAGCTGAAGGGCATCGACTTCAAGGAGGACGGCAACATCCTGGGGCACAAGCTGGAGTACAACTACAACAGCCACAACGTCTATATCATGGCCGACAAGCAGAAGAACGGCATCAAGGTGAACTTCAAGATCCGCCACAACATCGAGGACGGCAGCGTGCAGCTCGCCGACCACTACCAGCAGAACACCCCCATCGGCGACGGCCCCGTGCTGCTGCCCGACAACCACTACCTGAGCACCCAGTCCGCCCTGAGCAAAGACCCCAACGAGAAGCGCGATCACATGGTCCTGCTGGAGTTCGTGACCGCCGCCGGGATCACTCTCGGCATGGACGAGCTGTACAAGGGTGGCGGTGGAAGTATCGACTGCCGATACGATCGATACCGAGATTCTCTGAGGACCGTGGGCTTTCTGCTACATCTATCCATCGGTCGTAGGATCCATGATCGATACATCAATCGCTCGAATCACAGCGTCGCACTACACGAACTAAATGTACTTGAGATACGTCTATGTGTGCGCCAGAAGTGCCTTGTACTAAGCTGTTCGATACGAACCATCGATCGTTCTACGATCGATTGGTCATACAATCGATCGCTACATGAGCTTGTCAATTCACTCGACGGTGGAGGTGGAAGTGGTGGAGGTGGAAGTGGTGGAGGTGGAAGTGGTGGAGGTGGAAGTGGTGGAGGTGGAAGTGGTGGAGGTGGAAGTGGTGGAGGTAGTATCGACTGCCGATACGATCGATACCGAGATTCTCTGAGGACCGTGGGCTTTCTGCTACATCTATCCATCGGTCGTAGGATCCATGATCGATACATCAATCGCTCGAATCACAGCGTCGCACTACACGAACTAAATGTACTTGAGATACGTCTATGTGTGCGCCAGAAGTGCCTTGTACTAAGCTGTTCGATACGAACCATCGATCGTTCTACGATCGATTGGTCATACAATCGATCGCTACATGAGCTTGTCAATTCACTCGACGGTGGAGGTGGAAGTGGTGGAGGTGGAAGTGGTGGAGGTGGAAGTGGTGGAGGTGGAAGTGGTGGAGGTGGAAGTGGTGGAGGTGGAAGTGGTGGAGGTAGTATCGACTGCCGATACGATCGATACCGAGATTCTCTGAGGACCGTGGGCTTTCTGCTACATCTATCCATCGGTCGTAGGATCCATGATCGATACATCAATCGCTCGAATCACAGCGTCGCACTACACGAACTAAATGTACTTGAGATACGTCTATGTGTGCGCCAGAAGTGCCTTGTACTAAGCTGTTCGATACGAACCATCGATCGTTCTACGATCGATTGGTCATACAATCGATCGCTACATGAGCTTGTCAATTCACTCGACGGTGGAGGTGGAAGTGGTGGAGGTGGAAGTGGTGGAGGTGGAAGTGGTGGAGGTGGAAGTGGTGGAGGTGGAAGTGGTGGAGGTGGAAGTGGTGGAGGTGGAAGTATCGACTGCCGATACGATCGATACCGAGATTCTCTGAGGACCGTGGGCTTTCTGCTACATCTATCCATCGGTCGTAGGATCCATGATCGATACATCAATCGCTCGAATCACAGCGTCGCACTACACGAACTAAATGTACTTGAGATACGTCTATGTGTGCGCCAGAAGTGCCTTGTACTAAGCTGTTCGATACGAACCATCGATCGTTCTACGATCGATTGGTCATACAATCGATCGCTACATGAGCTTGTCAATTCACTCGACTGA**

**The insertion sequence of 2451bp：**

**ATGGTGAGCAAGGGCGAGGAGCTGTTCACCGGGGTGGTGCCCATCCTGGTCGAGCTGGACGGCGACGTAAACGGCCACAAGTTCAGCGTGTCCGGCGAGGGCGAGGGCGATGCCACCTACGGCAAGCTGACCCTGAAGTTCATCTGCACCACCGGCAAGCTGCCCGTGCCCTGGCCCACCCTCGTGACCACCCTGACCTACGGCGTGCAGTGCTTCAGCCGCTACCCCGACCACATGAAGCAGCACGACTTCTTCAAGTCCGCCATGCCCGAAGGCTACGTCCAGGAGCGCACCATCTTCTTCAAGGACGACGGCAACTACAAGACCCGCGCCGAGGTGAAGTTCGAGGGCGACACCCTGGTGAACCGCATCGAGCTGAAGGGCATCGACTTCAAGGAGGACGGCAACATCCTGGGGCACAAGCTGGAGTACAACTACAACAGCCACAACGTCTATATCATGGCCGACAAGCAGAAGAACGGCATCAAGGTGAACTTCAAGATCCGCCACAACATCGAGGACGGCAGCGTGCAGCTCGCCGACCACTACCAGCAGAACACCCCCATCGGCGACGGCCCCGTGCTGCTGCCCGACAACCACTACCTGAGCACCCAGTCCGCCCTGAGCAAAGACCCCAACGAGAAGCGCGATCACATGGTCCTGCTGGAGTTCGTGACCGCCGCCGGGATCACTCTCGGCATGGACGAGCTGTACAAGGGTGGCGGTGGAAGTATCGACTGCCGATACGATCGATACCGAGATTCTCTGAGGACCGTGGGCTTTCTGCTACATCTATCCATCGGTCGTAGGATCCATGATCGATACATCAATCGCTCGAATCACAGCGTCGCACTACACGAACTAAATGTACTTGAGATACGTCTATGTGTGCGCCAGAAGTGCCTTGTACTAAGCTGTTCGATACGAACCATCGATCGTTCTACGATCGATTGGTCATACAATCGATCGCTACATGAGCTTGTCAATTCACTCGACGGTGGAGGTGGAAGTGGTGGAGGTGGAAGTGGTGGAGGTGGAAGTGGTGGAGGTGGAAGTGGTGGAGGTGGAAGTGGTGGAGGTGGAAGTGGTGGAAGTATCGACTGCCGATACGATCGATACCGAGATTCTCTGAGGACCGTGGGCTTTCTGCTACATCTATCCATCGGTCGTAGGATCCATGATCGATACATCAATCGCTCGAATCACAGCGTCGCACTACACGAACTAAATGTACTTGAGATACGTCTATGTGTGCGCCAGAAGTGCCTTGTACTAAGCTGTTCGATACGAACCATCGATCGTTCTACGATCGATTGGTCATACAATCGATCGCTACATGAGCTTGTCAATTCACTCGACGGTGGAGGTGGAAGTGGTGGAGGTGGAAGTGGTGGAGGTGGAAGTGGTGGAGGTGGAAGTGGTGGAGGTGGAAGTGGTGGAGGTGGAAGTGGTGGAAGTATCGACTGCCGATACGATCGATACCGAGATTCTCTGAGGACCGTGGGCTTTCTGCTACATCTATCCATCGGTCGTAGGATCCATGATCGATACATCAATCGCTCGAATCACAGCGTCGCACTACACGAACTAAATGTACTTGAGATACGTCTATGTGTGCGCCAGAAGTGCCTTGTACTAAGCTGTTCGATACGAACCATCGATCGTTCTACGATCGATTGGTCATACAATCGATCGCTACATGAGCTTGTCAATTCACTCGACGGTGGAGGTGGAAGTGGTGGAGGTGGAAGTGGTGGAGGTGGAAGTGGTGGAGGTGGAAGTGGTGGAGGTGGAAGTGGTGGAGGTGGAAGTGGTGGAAGTATCGACTGCCGATACGATCGATACCGAGATTCTCTGAGGACCGTGGGCTTTCTGCTACATCTATCCATCGGTCGTAGGATCCATGATCGATACATCAATCGCTCGAATCACAGCGTCGCACTACACGAACTAAATGTACTTGAGATACGTCTATGTGTGCGCCAGAAGTGCCTTGTACTAAGCTGTTCGATACGAACCATCGATCGTTCTACGATCGATTGGTCATACAATCGATCGCTACATGAGCTTGTCAATTCACTCGACGGTGGAGGTGGAAGTGGTGGAGGTGGAAGTGGTGGAGGTGGAAGTGGTGGAGGTGGAAGTGGTGGAGGTGGAAGTGGTGGAGGTGGAAGTGGTGGAAGTATCGACTGCCGATACGATCGATACCGAGATTCTCTGAGGACCGTGGGCTTTCTGCTACATCTATCCATCGGTCGTAGGATCCATGATCGATACATCAATCGCTCGAATCACAGCGTCGCACTACACGAACTAAATGTACTTGAGATACGTCTATGTGTGCGCCAGAAGTGCCTTGTACTAAGCTGTTCGATACGAACCATCGATCGTTCTACGATCGATTGGTCATACAATCGATCGCTACATGAGCTTGTCAATTCACTCGACTGA**
