# Supplementary material for: Development of canine parvovirus-2-based recombinant pseudoviruses expression system: a potential vaccine platform
Source: Vet Res. 2026 Jun 18;57:110. doi: 10.1186/s13567-026-01789-9 (PMC13277292; doi:10.1186/s13567-026-01789-9)
Supplement: Supplementary file 2 — Additional file 2 Animal Welfare and Ethical Safeguards. [file 13567_2026_1789_MOESM2_ESM.docx]

**Additional File 2**

**Animal Welfare and Ethical Safeguards**

All animal experimental procedures have been reviewed and officially approved by the Animal Ethics Committee of Northwest A&F University, China (permit 20231106). The committee specifically evaluated and approved these aspects to ensure the animal welfare. During the study, all experiments were strictly performed in accordance with the protocol approved by the ethics committee, and no violations of animal ethics occurred.

The humane endpoint was strictly defined, including: (1) The body temperature exceeding 40.5℃ or below 37℃ for more than 48 hours; (2) The respiratory rate higher than 80 breaths per minute for more than 2 hours; (3) Severely listless mental state, with drowsiness and no response to external stimuli; (4) Severe neurological symptoms (e.g., limb paralysis, ataxia); (5) The weight loss exceeding 20% of the initial body weight for 3 consecutive days; (6) Severe diarrhea lasting for more than 24 hours, accompanied by loss of appetite and severe dehydration, and no improvement after symptomatic treatment. The Experimental animal meeting any of these endpoints will be euthanized humanely to prevent further suffering. The euthanasia protocol strictly adheres to the requirements of the American Veterinary Medical Association (AVMA). The operation was performed by the licensed veterinarian, as follows: Animals were first sedated with an intramuscular injection of xylazine (2 mg/kg), followed by the intravenous overdose of sodium pentobarbital (150 mg/kg) to ensure a painless and stress-free death.

Throughout the post-challenge period, we implemented the following measures to monitor and minimize animal suffering: (1) Monitoring measures: Monitor the health status of animals twice a day, recording their body temperature, appetite, water intake, fecal properties and mental state. Weigh the body weight once a day; (2) Supportive Care: The standardized supportive care was provided for animals to alleviate their suffering. This includes intravenous injection of lactated Ringer's solution to prevent dehydration and supply nutrition, as well as offering nutrient-fortified food. (3) Pain Management: The primary diseases were viral infections (CPV-2 and CDV), among which non-steroidal anti-inflammatory drugs can be contraindicated due to their impact on the gastrointestinal and hematological systems. We alleviated animal discomfort through supportive care, specifically maintaining appropriate temperature and humidity, providing soft padding, and feeding high-nutrition, palatable liquid or semi-liquid food.

The experimental design and result reporting of this study strictly followed the ARRIVE 2.0 guidelines. We have clearly described all relevant details in the revised manuscript, including sample size rationality, randomisation, blinding, statistical methods, and ethical statements. The virus challenge dose and vaccine immunization dose were determined through pre-experiment. A total of 25 4-week-old beagles were randomly divided into five groups, three of these groups of dogs were immunized with 1×10^6^ TCID_50_, 5×10^6^ TCID_50_, and 1×10^7^ TCID_50_ CPV-CDV pseudoviruses by intramuscular injection, respectively, one group dogs were immunized with a commercial vaccine containing live attenuated CPV-2 (1×10^5^ TCID_50_) and CDV (1×10^7^ TCID_50_), and the last one group dogs were injected with the same volume of PBS. All groups of dogs were immunized three times at 0 w, 3 w, and 6 w post immunization, and then challenged with 1×10^6^ TCID_50_ CPV-2 and 1×10^7^ TCID_50_ CDV at 8 w post immunization. Using blind methods for animal feeding management, sample collection, and testing.
