# Supplementary material for: Development of canine parvovirus-2-based recombinant pseudoviruses expression system: a potential vaccine platform
Source: Vet Res. 2026 Jun 18;57:110. doi: 10.1186/s13567-026-01789-9 (PMC13277292; doi:10.1186/s13567-026-01789-9)
Supplement: Supplementary file 3 — Additional file 3 Predicted B cell epitopes of the CPV VP2 and CDV H proteins. [file 13567_2026_1789_MOESM3_ESM.docx]

**Table S1. Predicted B cell epitopes of the CPV VP2 and CDV H proteins**

| **Source** | **Peptide Name** | **Peptide** | **Start** | **End** | **Length** |
| --- | --- | --- | --- | --- | --- |
| CPV-2 VP1/VP2 | VP 1 | PGNSLDQGEPTNPSD | 22 | 36 | 15 |
|  | VP 2 | TSRPSKPTKRSKPP | 105 | 118 | 14 |
|  | VP 3 | NLAPMSDGGVQ | 140 | 150 | 11 |
|  | VP 4 | AVRNERATGSGNGSG | 157 | 171 | 15 |
|  | VP 5 | NNQTEFKFLENG | 189 | 200 | 12 |
|  | VP 6 | VNNLDKTAVNGNMALDD | 227 | 243 | 17 |
|  | VP 7 | TLIPSHTGTSGTPTNIYHG | 360 | 378 | 19 |
|  | VP 8 | AEGGTNFGYIGVQQDKRRGVTQMG | 440 | 463 | 24 |
|  | VP 9 | GGAQTDENQAADGD | 505 | 518 | 14 |
|  | VP 10 | QKTTTTGETPER | 529 | 540 | 12 |
|  | VP 11 | LTNEYDPDASANM | 649 | 661 | 13 |
|  | VP 12 | WNPIQQMSINVDNQFNYV | 688 | 705 | 18 |
| CDV H | H 1 | GAFYKDNARANS | 9 | 20 | 12 |
|  | H 2 | VTEEQGGRRP | 26 | 35 | 10 |
|  | H 3 | LKEDMEKSEAVHHQV | 74 | 88 | 15 |
|  | H 4 | LSALSGGRGDIFPPYRCSG | 172 | 190 | 19 |
|  | H 5 | YHDSNGSQDGI | 305 | 315 | 11 |
|  | H 6 | VSEKQEEQKNCLES | 367 | 380 | 14 |
|  | H 7 | GMDYYESPLLDSG | 436 | 448 | 13 |
|  | H 8 | NKASRGDQFTVIPHV | 464 | 478 | 15 |
|  | H 9 | ADITNSTTSVENLVRIR | 583 | 599 | 17 |
