# Supplementary material for: Development of canine parvovirus-2-based recombinant pseudoviruses expression system: a potential vaccine platform
Source: Vet Res. 2026 Jun 18;57:110. doi: 10.1186/s13567-026-01789-9 (PMC13277292; doi:10.1186/s13567-026-01789-9)
Supplement: Supplementary file 4 — Additional file 4 Predicted CD8+ T cell immunity associated epitopes of CPV-2 VP1/VP2 and CDV H using NetMHCpan EL 4.1 and NetCTL 1.2 Server. The prediction was performed with the dog MHC allele (dog leukocyte antigen, DLA). [file 13567_2026_1789_MOESM4_ESM.docx]

**Table S2. Predicted CD8+ T cell immunity associated epitopes of CPV-2 VP1/VP2 and CDV H using NetMHCpan EL 4.1 and NetCTL 1.2 Server. The prediction was performed with the dog MHC allele (dog leukocyte antigen, DLA).**

| **Source** | **Peptide Name** | **Peptide** | **Start** | **End** | **Length** | **Tool** | **Allele** |
| --- | --- | --- | --- | --- | --- | --- | --- |
| CPV-2 VP1/VP2 | VP 1 | LSYQDKVSAFY | 2 | 12 | 11 | NetMHCpan | DLA-8850101, DLA-8803401, DLA-8850801 |
|  | VP 2 | QVSTSNMEF | 62 | 70 | 9 | NetCTL |  |
|  | VP 3 | QVIDVLTPLF | 87 | 96 | 10 | NetMHCpan |  |
|  | VP 4 | KLNEIKQFIL | 110 | 119 | 10 | NetMHCpan |  |
|  | VP 5 | LTAISDGVY | 220 | 228 | 9 | NetCTL |  |
|  | VP 6 | KTYLLVHDY | 230 | 238 | 9 | NetCTL |  |
|  | VP 7 | RVFEIGFIKR | 249 | 258 | 10 | NetMHCpan |  |
|  | VP 8 | CVDESTVLLY | 296 | 305 | 10 | NetCTL |  |
|  | VP 9 | KIHITNHRGF | 344 | 353 | 10 | NetMHCpan |  |
|  | VP 10 | ESACQRKSY | 379 | 387 | 9 | NetCTL |  |
|  | VP 11 | ILNGDGMDY | 431 | 439 | 9 | NetCTL |  |
|  | VP 12 | LLDSGWLTI | 444 | 452 | 9 | NetCTL |  |
|  | VP 13 | STVIPHVFTF | 472 | 481 | 10 | NetMHCpan |  |
|  | VP 14 | VVLPTQNFRY | 511 | 520 | 10 | NetMHCpan |  |
|  | VP 15 | YTYPFRLTTK | 547 | 556 | 10 | NetMHCpan |  |
| CDV H | H 1 | RGLVPPGYKY | 10 | 19 | 10 | NetMHCpan | DLA-8850101, DLA-8803401, DLA-8850801 |
|  | H 2 | YLRSGKNPYLY | 49 | 59 | 11 | NetCTL |  |
|  | H 3 | KRSKPPPHIF | 113 | 122 | 10 | NetMHCpan |  |
|  | H 4 | LVDANAWGV | 254 | 262 | 9 | NetCTL |  |
|  | H 5 | TMSELHLVSF | 275 | 284 | 10 | NetMHCpan |  |
|  | H 6 | ATQPPTKVY | 300 | 308 | 9 | NetCTL |  |
|  | H 7 | AAMRSETLGFY | 331 | 341 | 11 | NetMHCpan |  |
|  | H 8 | KPTIPTPWRY | 344 | 353 | 10 | NetMHCpan |  |
|  | H 9 | TSGTPTNIY | 368 | 376 | 9 | NetCTL |  |
|  | H 10 | GTDPDDVQF | 378 | 386 | 9 | NetCTL |  |
|  | H 11 | QAADGDPRY | 513 | 521 | 9 | NetCTL |  |
|  | H 12 | IAHQDTGRY | 544 | 552 | 9 | NetCTL |  |
